# Increasing trend in rhegmatogenous retinal detachment in Korea from 2004 to 2015

**DOI:** 10.1186/s12886-021-02157-1

**Published:** 2021-11-26

**Authors:** Jun Young Park, Seoung Jun Byun, Se Joon Woo, Kyu Hyung Park, Sang Jun Park

**Affiliations:** 1 Department of Ophthalmology, Seoul National University College of Medicine, Seoul National University Bundang Hospital, 173-82 Gumi-ro, Bundang-gu, Seongnam-si, Gyeonggi-do South Korea; 2 Department of Ophthalmology, Eulji University School of Medicine, Uijeongbu Eulji Medical Center, 712 Dongil-ro, Uijeongbu-si, Gyeonggi-do Uijeongbu, South Korea

**Keywords:** Incidence, Rhegmatogenous retinal detachment, Trend

## Abstract

**Background:**

To determine the 12-year incidence of and trends in rhegmatogenous retinal detachment (RRD) requiring surgery in Korea.

**Methods:**

This was a nationwide, population-based, retrospective study. We identified 53,179 patients with incident RRD requiring surgery using the Korean National Health Claims Database from 2004 to 2015. We estimated the crude incidence rates and age- and sex-standardized incidence rates per 100,000 person-years in each year during the study period. A joinpoint regression analysis was performed to determine the trend.

**Results:**

The average annual incidence rate was 9.78 (95% CI: 9.70–9.86). Male patients showed an incidence rate (10.68 [95% CI: 10.57–10.80]) 1.20 times that of female patients (8.87 [95% CI: 8.76–8.98]). The incidence showed a bimodal distribution; the highest peak was in the 60–64 year age group (23.77 [95% CI: 23.18–24.35]) and the second peak was in the 20–24 year age group (7.68 [95% CI: 7.41–7.95]). An increasing trend of RRD incidence was observed in the total population throughout the study period using joinpoint analysis (annual percentage change [APC], 2.05; 95% CI: 0.7–3.4). The increasing trend was more prominent among individuals aged under 50 years (APC, 3.44; 95% CI: 2.3–4.6), while among those aged 50 years or above, the increasing incidence was observed only in male patients.

**Conclusions:**

In Korea, the incidence of RRD has increased recently. People in the < 50 year age group accounted for the major part of this significant increase, which is related to the increasing incidence of myopia in the young generation in Asia.

**Supplementary Information:**

The online version contains supplementary material available at 10.1186/s12886-021-02157-1.

## Brief summary statement

An increasing trend in the incidence of rhegmatogenous retinal detachment was observed using data from Korean National Health Insurance service during the 12-year-study period from 2004 to 2015. People younger than 50 years were considered to account for a major portion of the increase in RRD cases.

## Background

Rhegmatogenous retinal detachment (RRD) is potentially a vision-threatening disease and one of the major causes of legal blindness. In the case of non-extensive RRD, pneumatic retinopexy and/or barrier laser photocoagulation can be successfully applied on an outpatient basis [1]. However, all RRD patients requiring surgical procedures such as scleral buckling, encircling, and pars plana vitrectomy are directly exposed to the risk of blindness, and it is necessary to accurately understand the incidence trend of RRD requiring surgery. Previous studies have reported that the annual incidence rate of RRD ranged from 6.8 to 18.2/100,000 persons with high variations over time and region [2–5]. In Asian studies, the reported annual incidence rates were 8, 14.4, 10.5, 10.4, and 10.4/100,000 for Beijing, Shanghai, Singapore, Japan, and Korea, respectively [6–10]. Currently, in Taiwan, the annual incidence of RRD is 16.40 per 100,000 persons according to data from the Taiwan National Health Insurance Research Database (2000–2012), with a younger mean age than most Western countries. A younger age at onset can be associated with retinal detachment in patients with high myopia [11]. However, no significant trend of RRD incidence was found throughout the study period. On the other hand, Nielsen et al. reported that the incidence of RRD is mainly increasing in the Caucasian population using the Danish National Patient Registry data from 2000 to 2016 [12] and suggested that the observed increase is primarily driven by male patients aged 50 years or older. Although not a nationwide cohort study, Redmer et al. also reported an increase in primary RRD incidence in the Dutch population with a simultaneous myopic shift [13].

Age, sex, myopia, pseudophakia, and a positive family history are well-known risk factors for RRD [14]. Particularly, among the risk factors, the prevalence of myopia is increasing worldwide [15]. In Asia, this is a relatively recent phenomenon that is mainly seen in the younger segment of the population. We wondered if RRD incidence is increasing in Korea over the recent decades, and if the main cause of the development in the incidence of RRD is related to the increase in myopia in the younger generation in Northeast Asia. The purpose of this study was to estimate the nationwide 12-year incidence rates of RRD requiring surgery and its trend from 2002 to 2015. To the best of our knowledge, no study has estimated the trend of RRD incidence in Asia over 10 years in a nationwide population.

## Methods

South Korea has a government-led, compulsory national health insurance scheme, the National Health Insurance Service (NHIS), which covers the entire South Korean population (approximately 50 million people). We obtained longitudinal data from 2002 to 2015 from the NHIS database, which includes all information regarding diagnoses, procedures, prescription records, demographic information, and direct medical costs. Detailed information regarding the NHIS and the database has been published elsewhere [16, 17], and the validation study showed the overall positive predictive value of the diagnosis to be 83.4% by comparing the diagnoses in the NHIS database with those in patient medical records. The NHIS database is accessible by researchers who have approved the research protocols by the official review committee in the NHIS. The study design was approved by the institutional review board of the Seoul National Bundang Hospital (IRB No. X-1710-429-903) and adhered to the tenets of the Declaration of Helsinki.

### Identification of patients

We identified incident RRD cases registered between 2004 and 2015 that required surgery as those with both the diagnostic code for RRD and the surgical code. The diagnostic code for RRD is H33.0 (RD with retinal break; H33.0, H33.00, H33.01, H33.02, H33.04, and H33.09). The surgical codes for RRD are S5130 (RD surgery including scleral buckling and encircling), S5121 (vitrectomy, total), and S5122 (vitrectomy, partial). We excluded cases that had both RRD diagnostic codes and surgical codes during the first 2 years of the study (2002 and 2003) to remove potential pre-existing cases of RRD. For this study, we excluded RRD cases that did not require surgical treatment. In addition, we excluded cases with the following diagnostic codes: H33.4 (traction detachment of the retina), H35.01 (exudative retinopathy, Coats’ disease), H35.1 (retinopathy of prematurity), H35.2 (other proliferative retinopathy, proliferative vitreoretinopathy), H44.0 (purulent endophthalmitis), H44.1 (other endophthalmitis: fungal endophthalmitis, virus-associated endophthalmitis, and sympathetic ophthalmia), H44.6 (retained intraocular foreign body, magnetic), H44.7 (retained intraocular foreign body, nonmagnetic), H45.1 (endophthalmitis in disease classified elsewhere), Q11.2 (microphthalmos), Q12 (congenital lens malformations), Q14 (congenital malformations of the posterior segment of the eye), S05.2 (ocular laceration and rupture with prolapse or loss of intraocular tissue), S05.3 (ocular laceration without prolapse or loss of intraocular tissue), S05.4 (penetrating wound of orbit with or without foreign body), S05.5 (penetrating wound of eyeball with foreign body), S05.6 (penetrating wound of eyeball without foreign body), and S05.7 (avulsion of eye). The index date was defined as the date of the earliest claim that corresponded to the above inclusion criteria. For cases that had two or more claims with diagnostic codes for RRD cases requiring surgery during the study period, the first claim in the database was defined as the incident time, and the patient was then counted as an incident case in that year.

### Statistical analysis

We calculated the crude incidence rates for each study year from 2004 to 2015 by dividing the number of RRD cases identified by the mid-year population (from resident registration data in Korea, available at: http://kosis.kr; accessed June 1, 2020) according to the 5-year age groups and sex. Standardized annual incidence rates for each study year were calculated using a direct method of standardization with stratifying age and gender based on the Korean census population in 2015 (available at: http://kosis.kr; accessed June 1, 2020) as the standard population. Using these standardized incidence rates, we calculated the weighted mean annual incidence rates during the study period (2004–2015) with 95% confidence intervals (CIs) based on the Poisson distribution. The units for all incidence data were per 100,000 person-years. The female-to-male ratio in each age group was also calculated.

To assess the trend of RRD incidence rates during the study period, we performed a joinpoint regression analysis using standardized incidence rates from 2004 to 2015. The joinpoint regression analysis is a segmented linear regression model used to investigate points where linear trends change significantly in magnitude or direction, which was developed by the Surveillance, Epidemiology, and End-Results (SEER) Program for checking the changes in cancer incidences [18]. SEER provides the software for the joinpoint regression analysis on its website (https://surveillance.cancer.gov/joinpoint; accessed 1 June 2020). The analysis starts with 0 joinpoints, which is straight line, and tests whether more joinpoints are statistically significant and must be added to the model up to that maximum number. The estimated trend is described by an annual percent change (APC) with a 95% CI. In addition, we performed joinpoint regression analyses after stratifying by sex and age groups (RRD patients aged < 60 years and those aged ≥ 60 years).

We then assessed the effect of birth cohorts on RRD occurrence and presented RRD incidence rates according to sequential 5-year birth cohorts beginning with the population born between 1910 and 1914 and ending with those born between 2005 and 2009. Since 12-year data on the RRD incidence from 2004 to 2015 were available, we were able compare the incidence rates in each age group among the three birth cohorts. All statistical analyses were performed using SAS (version 9.3; SAS Institute Inc., Cary, NC, USA) and R version 3.1.0 (The R Foundation for Statistical Computing, Vienna, Austria, http://www.R-project.org).

## Results

A total of 53,179 individuals (29,113 male, 54.7%) with RRD requiring surgery were included in this study during the 12-year study period. The numbers of cases were 2947 (5.5%) in 2004, 3487 (6.6%) in 2005, 3875 (7.3%) in 2006, 4145 (7.8%) in 2007, 4508 (8.5%) in 2008, 4460 (8.4%) in 2009, 4870 (9.2%) in 2010, 4555 (8.6%) in 2011, 4625 (8.7%) in 2012, 5037 (9.5%) in 2013, 5262 (9.9%) in 2014, and 5408 (10.2%) in 2015.

**Fig. 1:**
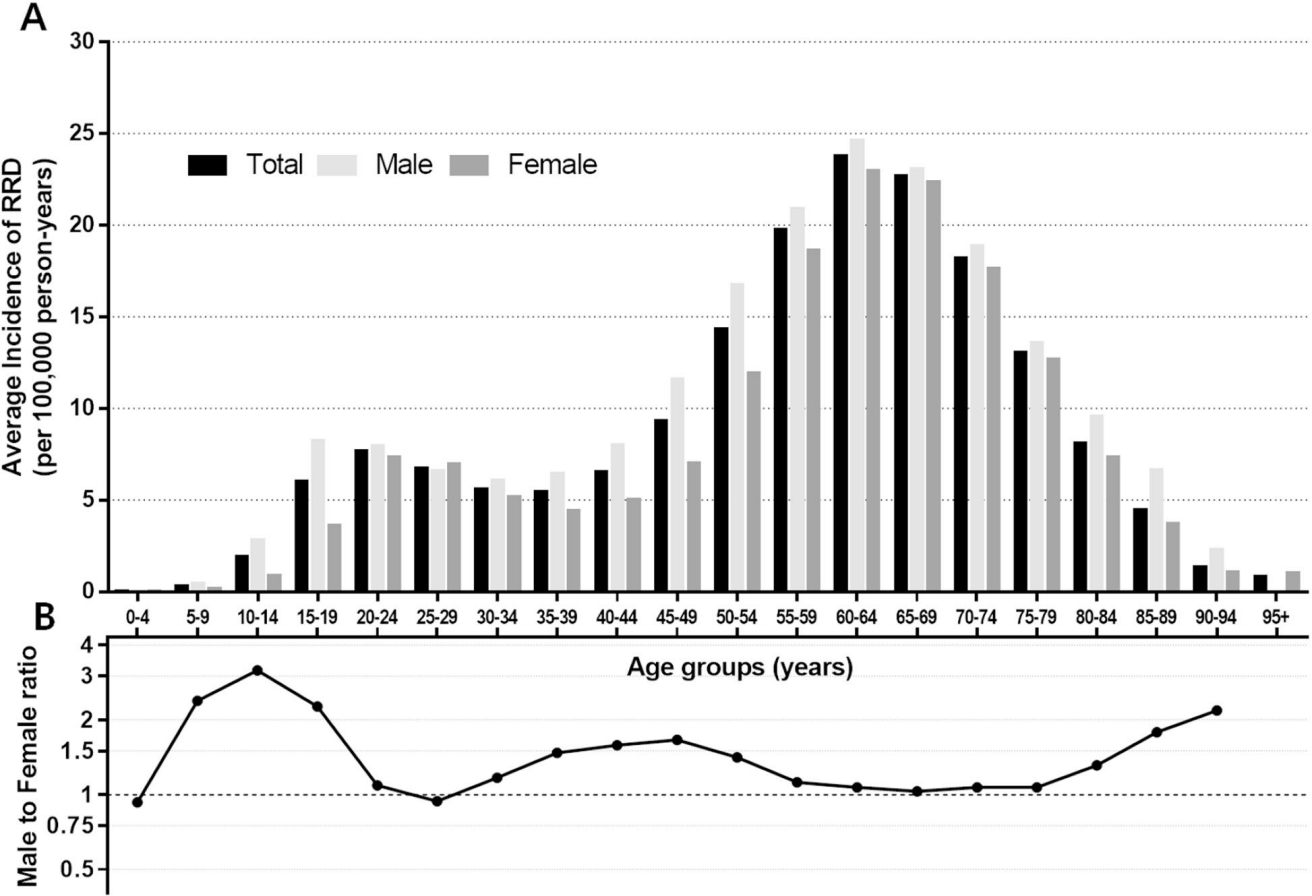
Average annual incidence (per 100,000 person-years) of rhegmatogenous retinal detachment (**A**) and the male-to-female ratio of the average annual incidence rhegmatogenous retinal detachment (**B**) in the Korean population by age group for the years 2004 to 2015

The standardized incidence rate of RRD cases requiring surgery was 9.78 (95% CI: 9.70–9.86) per 100,000 person-years during the study period; that in male and female patients was 10.68 per 100,000 person-years (95% CI: 10.57–10.80) and 8.87 per 100,000 person-years (95% CI: 8.76–8.98), respectively. The incidence of RRD in male patients was 1.20 times that in female patients (*p* < 0.001) (Table 1). RRD incidence according to age group showed a bimodal distribution; the highest peak was observed in the 60–64 year age group (23.77 [95% CI: 23.18–24.35]) and the second peak was observed in the 20–24 year age group (7.68 [95% CI: 7.41–7.95]) (Fig. 1A). The highest peaks were observed in the same age group, namely 60–64 years, in both male (24.62 [95% CI: 23.76–25.48]) and female patients (22.97 [95% CI: 22.17–23.77]), while the second peaks were observed in the 15–19 year age group (8.25 [95% CI: 7.87–8.64] in male patients and in the 20–24 year age group (7.34 [95% CI: 6.96–7.72] in female patients.

**Table 1:** Frequencies and Incidence Rates of Rhegmatogenous Retinal Detachment in Korean Population from 2004 to 2015

Age group (years)	Korean Population^a^ (*No.*)			Total		Male		Female		Male to female ratio
	Total	Male	Female	*No.*	Incidence^b^ (95% CI)	*No.*	Incidence (95% CI)	*No.*	Incidence (95% CI)	
0–4	2,235,397	1,147,126	1,088,271	10	0.04 (0.01–0.06)	5	0.03 (0.00–0.06)	5	0.04 (0.00–0.07)	0.93
5–9	2,252,950	1,162,087	1,090,863	104	0.32 (0.26–0.38)	75	0.44 (0.34–0.54)	29	0.18 (0.12–0.25)	2.38
10–14	2,418,360	1,257,902	1,160,458	740	1.91 (1.77–2.04)	576	2.84 (2.60–3.07)	164	0.90 (0.76–1.03)	3.16
15–19	3,170,545	1,657,722	1512,823	2443	6.05 (5.81–6.29)	1751	8.25 (7.87–8.64)	692	3.65 (3.37–3.92)	2.26
20–24	3,385,936	1,808,857	1,577,079	3145	7.68 (7.41–7.95)	1709	7.98 (7.60–8.36)	1436	7.34 (6.96–7.72)	1.09
25–29	3,027,896	1,581,887	1,446,009	2982	6.76 (6.52–7.00)	1490	6.58 (6.24–6.91)	1492	6.96 (6.61–7.32)	0.94
30–34	3,611,034	1,854,905	1,756,129	2753	5.63 (5.42–5.84)	1512	6.06 (5.75–6.36)	1241	5.18 (4.89–5.46)	1.17
35–39	3,783,589	1,927,388	1,856,201	2816	5.47 (5.26–5.67)	1700	6.48 (6.17–6.79)	1116	4.41 (4.15–4.67)	1.47
40–44	4,215,921	2,142,101	2,073,820	3454	6.56 (6.34–6.78)	2147	8.00 (7.66–8.34)	1307	5.06 (4.79–5.34)	1.58
45–49	4,266,941	2,151,070	2,115,871	4729	9.33 (9.07–9.60)	2987	11.62 (11.20–12.04)	1742	7.00 (6.67–7.33)	1.66
50–54	4145,976	2,094,318	2,051,658	6374	14.36 (14.01–14.72)	3752	16.77 (16.24–17.31)	2622	11.91 (11.45–12.36)	1.41
55–59	3,863,095	1,922,796	1,940,299	6774	19.76 (19.29–20.23)	3566	20.92 (20.23–21.60)	3208	18.62 (17.98–19.27)	1.12
60–64	2,758,941	1,348,273	1,410,668	6296	23.77 (23.18–24.35)	3159	24.62 (23.76–25.48)	3137	22.97 (22.17–23.77)	1.07
65–69	2,117,875	1,015,463	1,102,412	5079	22.69 (22.07–23.32)	2384	23.06 (22.14–23.99)	2695	22.36 (21.51–23.20)	1.03
70–74	1,760,932	789,607	971,325	3244	18.20 (17.57–18.82)	1435	18.90 (17.92–19.88)	1809	17.62 (16.80–18.43)	1.07
75–79	1,356,014	550,684	805,330	1548	13.05 (12.40–13.70)	603	13.57 (12.49–14.65)	945	12.68 (11.88–13.49)	1.07
80–84	810,891	275,462	535,429	537	8.10 (7.42–8.78)	203	9.59 (8.27–10.90)	334	7.34 (6.55–8.12)	1.31
85–89	371,527	98,367	273,160	136	4.50 (3.74–5.26)	54	6.65 (4.87–8.42)	82	3.73 (2.92–4.54)	1.78
90–94	124,111	28,565	95,546	13	1.34 (0.61–2.07)	5	2.30 (0.28–4.32)	8	1.06 (0.32–1.79)	2.18
95+	27,732	5259	22,473	2	0.85 (0.00–2.04)	NE	NE	2	1.05 (0.00–2.50)	0.00
Total	49,705,663	24,819,839	24,885,824	53,179	9.78 (9.70–9.86)	29,113	10.68 (10.57–10.80)	24,066	8.87 (8.76–8.98)	1.20

*CI* confidence interval, *NE* no estimate

^a^Korean population was represented by the census population in 2015 according to the Korean Statistical Information Service

^b^The unit for incidence rate is per 100,000 person-years

The incidence of RRD decreased with increasing age in both men and women aged ≥ 65 years. Male patients had a higher RRD incidence rate than female patients in all age groups except for patients aged < 5 years and 25–29 years. The graph shows that the female-to-male ratio ranged from 0.93 to 3.16, with the highest peak observed in the 10–14 year age group (Fig. 1B).

**Fig. 2:**
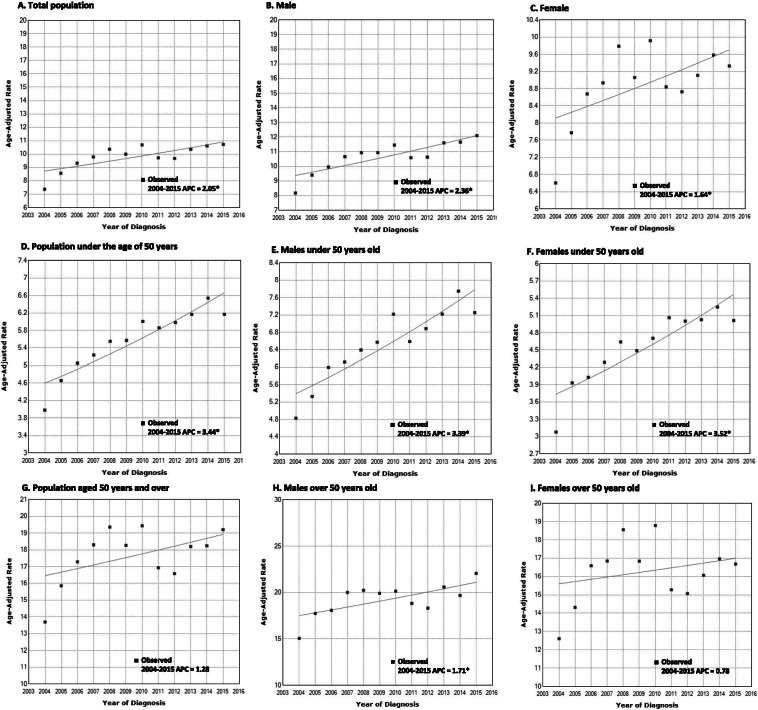
Joinpoint regression analysis of trends in rhegmatogenous retinal detachment (RRD) **A.** The significant increasing incidence of RRD was observed from 2004 to 2015 for the entire population. **B, and C.** Significant increasing trend was observed in both male and female patients. **D.** Incidence rates of RRD increased in the population aged < 50 years, **E, and F.** Both male and female patients aged < 50 years demonstrated an increasing trend. **G**. No significant trend was observed for patients aged ≥ 50 years from 2004 to 2015. **H.** Male patients over the age of 50 demonstrated a significant increasing trend. **I.** No significant trend was observed in females over the age of 50 years. ^*^p < 0.05, APC; annual percent change

The standardized annual incidence rates of surgically treated RRD per 100,000 persons ranged from 7.39 to 10.71 in the 12-year study period (Table 2). The standardized annual incidence rates showed an increasing trend throughout the 12-year study period. This overall increasing trend was observed for both sexes. The joinpoint regression analysis showed an obvious increasing trend (APC = 2.05; 95% CI: 0.7–3.4; *p* < 0.01), which was also observed in both male (APC = 2.36; 95% CI: 1.3–3.4; *p* < 0.01) and female patients (APC = 1.64; 95% CI; 0.0–3.3; *p* < 0.01) (Fig. 2A, B, and C). When the RRD patients were divided in reference to the age of 50 years, a steeper increase in RRD incidence rates was shown among patients aged < 50 years (APC = 3.44; 95% CI: 2.3–4.6; *p* < 0.01); this was also observed in both male (APC = 3.39; 95% CI: 2.3–4.5; *p* < 0.01) and female patients (APC = 3.52; 95% CI: 2.2–4.9; *p* < 0.01) (Fig. 2F). The individuals aged ≥ 50 years partially showed a significant increase from 2004 to 2007 (APC = 8.36; 95% CI: 6.1–10.6; *p* < 0.01) when 2 joinpoints were applied, however, there was no statistically significant trend in terms of the entire period (Fig. 2G). When limited to men aged ≥ 50 years, the incidence rate showed an increasing trend (APC = 1.71; 95% CI: 0.4–3.0; *p* < 0.01), but not when limited to female patients (Fig. 2H and I).

**Table 2:** Yearly Age-standardized Incidence Rates (per 100,000 persons) of Rhegmatogenous Retinal Detachment from 2004 to 2015

	2004	2005	2006	2007	2008	2009	2010	2011	2012	2013	2014	2015
0–4	0.08	0.08	0.00	0.04	0.04	0.00	0.00	0.04	0.09	0.00	0.00	0.04
	(−0.04–0.19)	(−0.04–0.19)	(0.00–0.00)	(−0.04–0.13)	(−0.04–0.13)	(0.00–0.00)	(0.00–0.00)	(−0.04–0.13)	(−0.04–0.21)	(0.00–0.00)	(0.00–0.00)	(−0.04–0.13)
5–9	0.15	0.24	0.50	0.36	0.35	0.22	0.47	0.42	0.30	0.17	0.35	0.30
	(−0.01–0.31)	(0.04–0.44)	(0.21–0.79)	(0.11–0.61)	(0.10–0.59)	(0.03–0.41)	(0.18–0.75)	(0.15–0.68)	(0.07–0.52)	(0.00–0.34)	(0.10–0.59)	(0.08–0.53)
10–14	1.34	1.56	1.97	1.80	2.46	2.62	2.00	1.66	2.33	1.39	1.83	1.84
	(0.88–1.81)	(1.07–2.06)	(1.41–2.53)	(1.26–2.33)	(1.84–3.09)	(1.98–3.27)	(1.43–2.56)	(1.14–2.17)	(1.72–2.94)	(0.92–1.86)	(1.29–2.37)	(1.30–2.38)
15–19	4.40	5.00	6.20	5.41	5.51	5.83	7.70	6.33	6.16	6.45	6.94	6.45
	(3.67–5.13)	(4.22–5.78)	(5.33–7.06)	(4.60–6.21)	(4.69–6.33)	(4.99–6.67)	(6.74–8.67)	(5.46–7.21)	(5.29–7.02)	(5.56–7.33)	(6.03–7.86)	(5.57–7.34)
20–24	5.31^b^	6.42	6.83	7.54	7.63	7.04	9.05	8.01	10.16	8.84	8.42	7.70
	(4.53–6.08)	(5.57–7.27)	(5.95–7.71)	(6.61–8.46)	(6.70–8.56)	(6.14–7.93)	(8.03–10.06)	(7.06–8.96)	(9.08–11.23)	(7.84–9.84)	(7.44–9.39)	(6.77–8.64)
25–29	5.04	5.29	5.96	6.85	6.76	6.70	7.76	8.42	7.75	7.03	6.83	7.19
	(4.24–5.84)	(4.47–6.10)	(5.09–6.83)	(5.92–7.78)	(5.83–7.69)	(5.78–7.63)	(6.77–8.75)	(7.38–9.45)	(6.76–8.74)	(6.08–7.97)	(5.90–7.76)	(6.23–8.14)
30–34	3.77	4.64	4.60	4.96	5.59	5.86	6.48	6.66	6.04	6.44	7.00	6.01
	(3.13–4.40)	(3.94–5.34)	(3.90–5.30)	(4.24–5.69)	(4.82–6.36)	(5.07–6.65)	(5.65–7.31)	(5.81–7.50)	(5.24–6.84)	(5.61–7.26)	(6.14–7.87)	(5.21–6.81)
35–39	4.11	4.05	5.06	5.40	5.37	5.29	5.23	6.06	6.02	6.26	6.63	6.49
	(3.47–4.76)	(3.41–4.69)	(4.35–5.78)	(4.66–6.14)	(4.63–6.11)	(4.56–6.03)	(4.50–5.96)	(5.28–6.84)	(5.24–6.80)	(5.47–7.06)	(5.81–7.45)	(5.68–7.31)
40–44	4.74	5.36	5.32	5.84	6.24	7.13	7.36	6.32	6.51	7.74	8.33	7.59
	(4.08–5.40)	(4.66–6.06)	(4.63–6.02)	(5.11–6.57)	(5.49–7.00)	(6.33–7.94)	(6.54–8.18)	(5.56–7.08)	(5.74–7.28)	(6.90–8.58)	(7.46–9.21)	(6.76–8.42)
45–49	6.72	8.84	8.98	8.81	9.83	9.20	8.32	8.89	8.77	10.39	11.63	11.20
	(5.95–7.50)	(7.95–9.74)	(8.08–9.88)	(7.92–9.70)	(8.88–10.77)	(8.29–10.11)	(7.46–9.19)	(7.99–9.78)	(7.88–9.65)	(9.42–11.36)	(10.61–12.65)	(10.19–12.20)
50–54	11.05	13.16	13.07	13.38	15.04	14.61	15.34	13.58	13.46	15.85	14.95	16.80
	(10.03–12.06)	(12.05–14.26)	(11.97–14.17)	(12.26–14.49)	(13.86–16.22)	(13.44–15.77)	(14.15–16.54)	(12.46–14.71)	(12.34–14.57)	(14.64–17.06)	(13.77–16.12)	(15.55–18.04)
55–59	15.31	18.98	18.69	22.27	21.12	18.95	21.03	18.06	18.69	20.14	21.28	20.99
	(14.07–16.54)	(17.61–20.35)	(17.33–20.06)	(20.78–23.76)	(19.67–22.57)	(17.58–20.33)	(19.58–22.48)	(16.72–19.40)	(17.33–20.05)	(18.72–21.55)	(19.83–22.74)	(19.54–22.43)
60–64	**19.12**^a^	**21.01**	22.73	22.96	25.60	**25.73**	**27.28**	**24.55**	**23.15**	22.82	**24.03**	**25.37**
	(17.49–20.75)	(19.30–22.72)	(20.95–24.51)	(21.17–24.75)	(23.71–27.49)	(23.84–27.63)	(25.33–29.23)	(22.70–26.39)	(21.36–24.95)	(21.03–24.60)	(22.20–25.86)	(23.49–27.25)
65–69	18.10	20.74	**23.60**	**23.22**	**27.27**	22.66	24.65	21.06	20.15	**23.18**	22.75	23.94
	(16.29–19.91)	(18.80–22.68)	(21.53–25.67)	(21.17–25.27)	(25.05–29.49)	(20.63–24.69)	(22.53–26.76)	(19.11–23.02)	(18.24–22.06)	(21.12–25.23)	(20.72–24.78)	(21.86–26.02)
70–74	13.73	16.34	18.69	20.94	20.20	19.87	19.42	16.61	17.40	18.86	17.14	18.29
	(12.00–15.46)	(14.45–18.23)	(16.67–20.71)	(18.80–23.08)	(18.10–22.30)	(17.78–21.95)	(17.36–21.48)	(14.71–18.52)	(15.45–19.35)	(16.84–20.89)	(15.21–19.08)	(16.29–20.29)
75–79	10.58	9.48	14.09	13.85	14.00	14.61	14.81	11.55	11.59	12.56	13.67	14.41
	(8.85–12.31)	(7.84–11.12)	(12.09–16.09)	(11.87–15.83)	(12.01–16.00)	(12.57–16.64)	(12.76–16.86)	(9.75–13.36)	(9.78–13.40)	(10.67–14.45)	(11.70–15.64)	(12.39–16.43)
80–84	3.58	4.10	8.57	7.40	9.22	9.68	10.14	9.36	6.47	10.04	8.25	8.06
	(2.27–4.88)	(2.71–5.49)	(6.56–10.59)	(5.53–9.27)	(7.13–11.31)	(7.54–11.83)	(7.95–12.33)	(7.26–11.47)	(4.72–8.22)	(7.86–12.22)	(6.28–10.23)	(6.11–10.02)
85–89	1.23	2.87	3.71	5.37	2.68	3.63	3.88	6.26	3.50	3.92	5.39	7.94
	(0.10–2.35)	(1.14–4.59)	(1.75–5.67)	(3.01–7.72)	(1.02–4.35)	(1.70–5.57)	(1.87–5.88)	(3.72–8.81)	(1.60–5.40)	(1.91–5.94)	(3.03–7.75)	(5.07–10.81)
90–94	3.93	1.77	1.66	0.00	0.00	0.00	3.91	2.35	1.05	0.00	0.86	1.61
	(0.44–7.42)	(−0.57–4.11)	(−0.61–3.93)	(0.00–0.00)	(0.00–0.00)	(0.00–0.00)	(0.43–7.39)	(−0.35–5.05)	(−0.75–2.85)	(0.00–0.00)	(−0.77–2.48)	(−0.62–3.84)
95+	0.00	0.00	0.00	0.00	0.00	5.49	0.00	4.70	0.00	0.00	0.00	0.00
	(0.00–0.00)	(0.00–0.00)	(0.00–0.00)	(0.00–0.00)	(0.00–0.00)	(−3.23–14.22)	(0.00–0.00)	(−3.37–12.76)	(0.00–0.00)	(0.00–0.00)	(0.00–0.00)	(0.00–0.00)
Total	7.39	8.58	9.31	9.80	10.36	10.00	10.68	9.72	9.67	10.35	10.62	10.71
	(7.15–7.63)	(8.32–8.84)	(9.05–9.58)	(9.52–10.07)	(10.07–10.64)	(9.72–10.27)	(10.39–10.97)	(9.44–9.99)	(9.40–9.95)	(10.07–10.64)	(10.33–10.90)	(10.42–11.00)

^a^Bold: the highest peak of incidence rate of rhegmatogenous retinal detachment across age groups

^b^Underbar: the second peak of incidence rate of rhegmatogenous retinal detachment across age groups

When they were divided in reference to the age of 40 years or 60 years, an increasing trend was observed both over and under 40 and under 60 years (APC = 1.70; 95% CI: 0.3–3.2; *p* < 0.01, APC = 3.43; 95% CI: 1.9–5.0; *p* < 0.01, and APC = 2.59; 95% CI: 1.5–3.7; *p* < 0.01, respectively; Additional file 1 [Fig. S1 A, B, and C]), but we could not find any trend in incidence rates among individuals aged ≥ 60 years (Additional file 1 [Fig. S1D]).

**Fig. 3:**
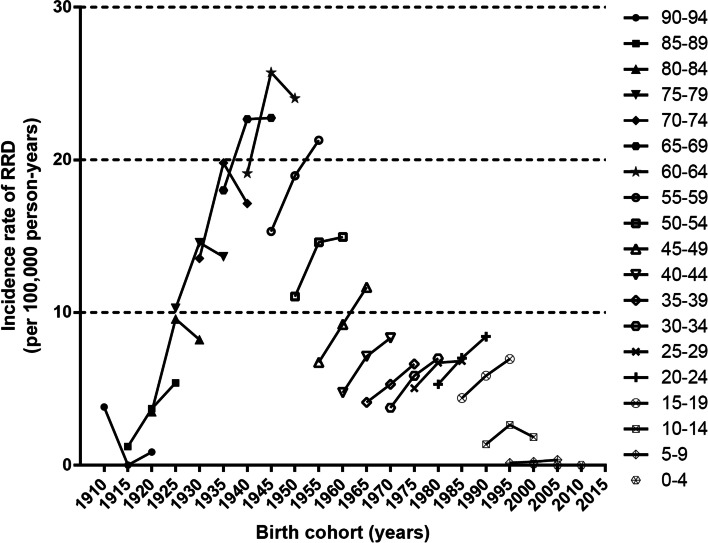
Birth cohort analysis of the annual incidence of rhegmatogenous retinal detachment (RRD) in each age group in 2004 (the first symbol in the same age group), 2009 (the second symbol in the same age group), and 2014 (the third symbol in the same age group). The incidence rate of RRD for the 15–59 year age group demonstrated an increase from 2004 to 2014. However, the incidence rate of RRD for the 60–89 year age group increased in 2009 compared with that in 2004, but decreased in 2014 compared with that in 2009

For the birth cohort born after 1945, corresponding to the 15–59 year age group, the incidence rate increased from 2004 to 2014, while for the 60–89 year age group, the incidence rate increased in 2009 compared with that in 2004, but decreased in 2014 compared with that in 2009 (Fig. 3). Detailed data on the number of RRD cases, estimated crude incidence rate, and estimated standardized incidence rates according to age groups and sex in each study year from 2004 to 2015 are provided in Additional file 2 (Tables S14–25, respectively).

## Discussion

This study demonstrated an increasing trend in the incidence of RRD cases requiring surgery during the last decades in Korea. The incidence of RRD over a 5-year period in Korea has been previously reported [10] but it is difficult to grasp the trends due to the relatively short study duration. Therefore, this study was designed over a 12-year study period to estimate the trend of incident RRD cases requiring surgery.

The yearly standardized incidence rate of RRD cases requiring surgery was 9.78/100,000 person-years (95% CI, 9.70–9.86) in this study (Table 1). Our report showed a lower annual RRD incidence rate than those in previous nationwide studies, which were reported to vary from 8 to 16.4 [2–10] and which might be due to the slightly different definitions of RRD occurrences, data collection method, and regional and ethnic differences in each study.

The male preference observed in the RRD incidence, a male to female ratio of 1.20 in this study (Fig. 1B), coincides with that in the other studies [6, 19]. The prevalence of trauma and symptomatic posterior vitreous detachment (PVD) complicated by a retinal tear, which is more common in male patients, may have an influence on the higher RRD risk in male patients [19]. The present study showed a bimodal distribution of the average RRD incidences with age over the 12-year study period; the highest peak being in the seventh decade of life and the second highest peak in the third decade (Fig. 1A). In each year from 2004 to 2015, the RRD incidence also demonstrated relatively constant highest and second highest peak age groups (Table 2). This result is consistent with our previous 5-year report in Korea and reports from other Asian countries on the incidence of RRD [6, 9, 10]. In the majority of studies, RRD incidence increases with age and shows the highest peak age of 50 to 79 years in both Caucasians and Asians [11, 19, 20]. PVD usually develops at 60 years of age and can play a critical role in the development of RRD. The volume of cataract extraction performed may partially affect the high RRD incidence rates in old age [12]; however, the second highest peak was clearly observed in Asians compared to Caucasians [19]. Myopia was more frequent and more severe in Asians than in Caucasians, and the myopia-induced PVD in the relatively young age groups may be associated with the second highest peak incidence of RRD [21, 22].

The RRD incidence showed an increasing trend from 2004 to 2015 in the total Korean population. With age stratification in reference to 50 years, an increase in RRD incidence in patients of both sexes aged < 50 years was also observed. Nielsen et al. reported that the RRD incidence is increasing, and this increase is primarily driven by male patients aged ≥ 50 years using the Danish National Patient Registry data from 2000 to 2016 [12]. However, in this study, APC in both male and female patients under 50 years of age (APC, 3.39; Fig. 2E and APC, 3.52; Fig. 2F, respectively) was much greater than that in male patients aged ≥ 50 years (APC, 1.71; Fig. 2H), which means that there is a trend with a similar increase in male patients aged ≥ 50 years in Asians compared to Caucasians, but among patients aged under 50 years, the RRD incidence in Asians showed a much greater increase than that in Caucasians. Myopia, a well-known risk factor for RRD, is increasing primarily in the young [15], especially in the Asian population. Korea is a country that has rapidly developed within a short period of time, and the increase in economic power and education level along with the body index, including body stature and body weight, can be attributed to the prevalence of myopia in the young age group, which is higher than that in the old age group [23]. Kim et al. and Lee et al. reported that the prevalence of myopia in Korea has increased over time based on the database of the Korean National Health and Nutrition Survey 2008–2011 and the Korean Military Manpower Administration 2009–2013, respectively [24, 25]. The prevalence of high myopia also has increased in younger individuals [24, 26]. In addition, myopic eyes demonstrated a gradual increase in axial length over time [27], which can induce an increase in the risk of RRD. Another major risk factor for RRD is a pseudophakic eye after cataract extraction [14, 28]. With improvements in the technical difficulty and surgical safety of phacoemulsification for cataract extraction, the number of phacoemulsification has been increasing year by year, and cataract extraction tends to be performed at an earlier stage in Korea [29]. Furthermore, in patients with high myopia, cataract surgery also tends to be performed at a younger age, and RRD as a postoperative complication after surgery is known to be more frequent [30].

In addition to the result of the joinpoint regression analysis, analysis of the 2004, 2009, and 2014 birth cohorts also showed a consistently increasing trend in RRD incidence in the same age group from 2004 to 2014 in the young generation under the age of 60 years in Korea (Fig. 3). In contrast, in participants aged ≥ 60 years, the RRD incidence rate increased in 2009 but decreased in 2014 on inspection of the 2004 birth cohort after 5 and 10 years. However, no statistically significant trend was found in people over 60 years old by the joinpoint regression analysis (Additional file 1 [Fig. S1D]). Partially, the population aged ≥ 50 years demonstrated a significant increasing trend from 2004 to 2007 unlike 2008–2014 and total period. Further analysis of this increase in early 2000s (2004 to 2007) may be necessary.

The limitation of this study is its nature based on claims database; hence, asymptomatic, unnoticeable or untreated RRD patients may exist by various factors such as old age, disease severity, socioeconomic status affecting healthcare service utilization, and systemic or ophthalmic comorbidity. These give rise to underestimate the RRD incidence. Furthermore, we were not able to access the hospital-based medical records of each patient for the severity and extent of RRD and the review of clinical data including myopia, pseudophakia, posterior vitreous detachment and other vision-devastating disease. Therefore, there is a limit to analyzing the association with RRD and these factors. In addition, since the database allowed for only 12-years of follow-up, we could only analyze three birth cohorts in each age group. Further investigation is needed to analyze the comprehensive birth cohort over a 40-years study period.

In conclusion, the current study showed an overall increasing trend in the incidence of RRD during the 12-year study period. As in Caucasians, the increase was observed in the total population in Korea. However, people younger than 50 years were considered to be important contributors to the increase in RRD incidence, unlike Caucasians. The increase in myopia and high myopia during the last few decades may play a role in this observation.

## Supplementary Information

**Additional file 1: Fig. S1.** Joinpoint regression analysis of trends in rhegmatogenous retinal detachment.

**Additional file 2: Supplemental Table 1.** Numbers According to Age Groups and Sex in the 2004 Midyear Population in Korea. **Supplemental Table 2.** Numbers According to Age Groups and Sex in the 2005 Midyear Population in Korea. **Supplemental Table 3.** Numbers According to Age Groups and Sex in the 2006 Midyear Population in Korea. **Supplemental Table 4.** Numbers According to Age Groups and Sex in the 2007 Midyear Population in Korea. **Supplemental Table 5.** Numbers According to Age Groups and Sex in the 2008 Midyear Population in Korea. **Supplemental Table 6.** Numbers According to Age Groups and Sex in the 2009 Midyear Population in Korea. **Supplemental Table 7.** Numbers According to Age Groups and Sex in the 2010 Midyear Population in Korea. **Supplemental Table 8.** Numbers According to Age Groups and Sex in the 2011 Midyear Population in Korea. **Supplemental Table 9.** Numbers According to Age Groups and Sex in the 2012 Midyear Population in Korea. **Supplemental Table 10.** Numbers According to Age Groups and Sex in the 2013 Midyear Population in Korea. **Supplemental Table 11.** Numbers According to Age Groups and Sex in the 2014 Midyear Population in Korea. **Supplemental Table 12.** Numbers According to Age Groups and Sex in the 2015 Midyear Population in Korea. **Supplemental Table 13.** Numbers according to age groups and sex in the 2015 Census of Population in Korea. **Supplemental Table 14.** Number of Cases of Rhegmatogenous Retinal Detachment (RRD) Requiring Surgery and Crude and Age- and Sex-Standardized Incidence Rate of RRD in 2004. **Supplemental Table 15.** Number of Cases of Rhegmatogenous Retinal Detachment (RRD) Requiring Surgery and Crude and Age- and Sex-Standardized Incidence Rate of RRD in 2005. **Supplemental Table 16.** Number of Cases of Rhegmatogenous Retinal Detachment (RRD) Requiring Surgery and Crude and Age- and Sex-Standardized Incidence Rate of RRD in 2006. **Supplemental Table 17.** Number of Cases of Rhegmatogenous Retinal Detachment (RRD) Requiring Surgery and Crude and Age- and Sex-Standardized Incidence Rate of RRD in 2007. **Supplemental Table 18.** Number of Cases of Rhegmatogenous Retinal Detachment (RRD) Requiring Surgery and Crude and Age- and Sex-Standardized Incidence Rate of RRD in 2008. **Supplemental Table 19.** Number of Cases of Rhegmatogenous Retinal Detachment (RRD) Requiring Surgery and Crude and Age- and Sex-Standardized Incidence Rate of RRD in 2009. **Supplemental Table 20.** Number of Cases of Rhegmatogenous Retinal Detachment (RRD) Requiring Surgery and Crude and Age- and Sex-Standardized Incidence Rate of RRD in 2010. **Supplemental Table 21.** Number of Cases of Rhegmatogenous Retinal Detachment (RRD) Requiring Surgery and Crude and Age- and Sex-Standardized Incidence Rate of RRD in 2011. **Supplemental Table 22.** Number of Cases of Rhegmatogenous Retinal Detachment (RRD) Requiring Surgery and Crude and Age- and Sex-Standardized Incidence Rate of RRD in 2012. **Supplemental Table 23.** Number of Cases of Rhegmatogenous Retinal Detachment (RRD) Requiring Surgery and Crude and Age- and Sex-Standardized Incidence Rate of RRD in 2013. **Supplemental Table 24.** Number of Cases of Rhegmatogenous Retinal Detachment (RRD) Requiring Surgery and Crude and Age- and Sex-Standardized Incidence Rate of RRD in 2014. **Supplemental Table 25.** Number of Cases of Rhegmatogenous Retinal Detachment (RRD) Requiring Surgery and Crude and Age- and Sex-Standardized Incidence Rate of RRD in 2015.

## Data Availability

The datasets used and analyzed during the current study are available from the corresponding authors on reasonable request.

## Abbreviations

APC: Annual percentage change
CI: Confidence interval
NHIS: National Health Insurance Service
PVD: Posterior vitreous detachment
RRD: Rhegmatogenous retinal detachment
