# Supplementary figures and images for: Increasing trend in rhegmatogenous retinal detachment in Korea from 2004 to 2015
Source: BMC Ophthalmol. 2021 Nov 26;21:406. doi: 10.1186/s12886-021-02157-1 (PMC8627102; doi:10.1186/s12886-021-02157-1)

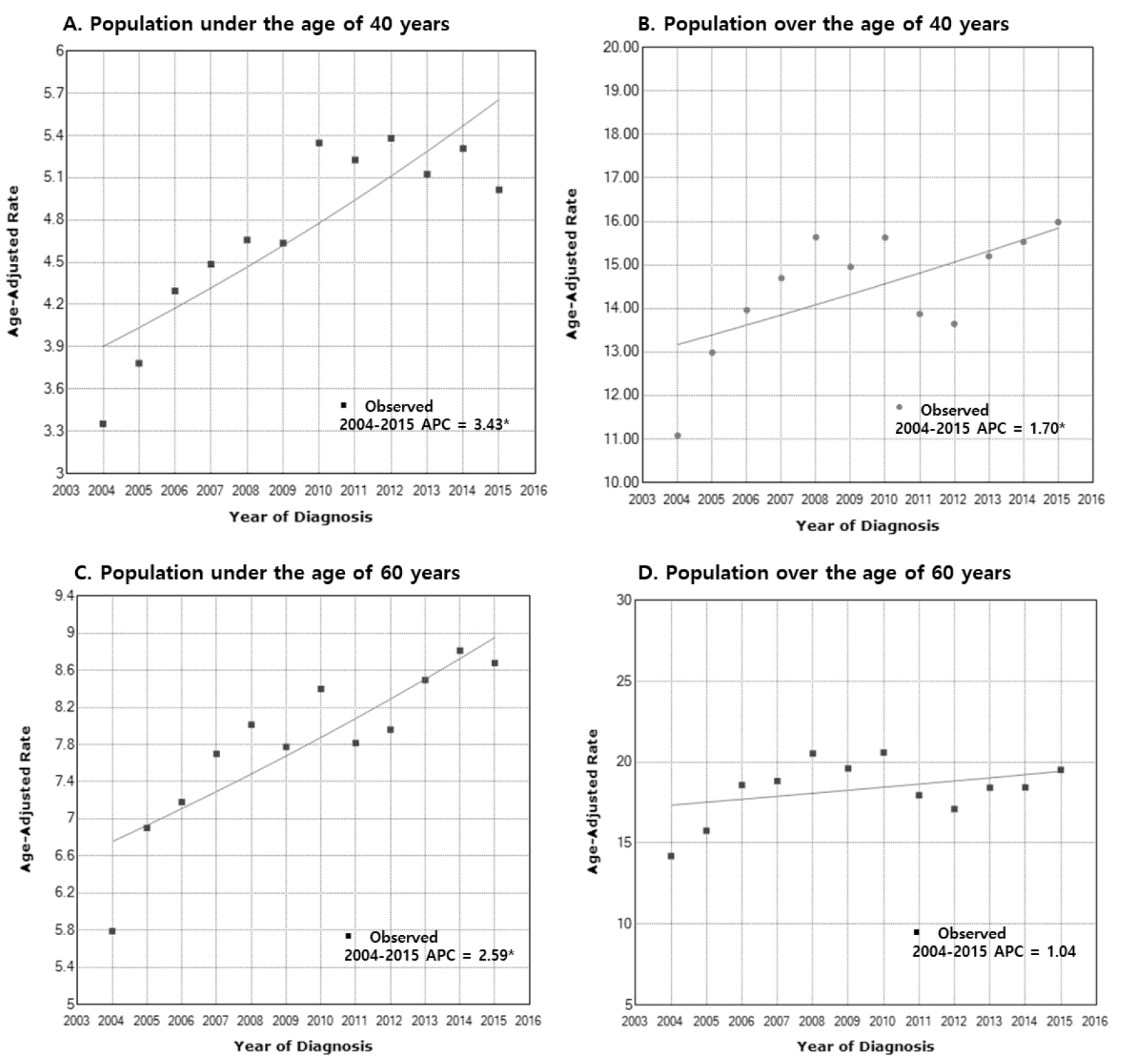

Supplement: Supplementary file 1 — Additional file 1: Fig. S1. Joinpoint regression analysis of trends in rhegmatogenous retinal detachment. [file 12886_2021_2157_MOESM1_ESM.tif]
