# Supplementary material for: Increasing trend in rhegmatogenous retinal detachment in Korea from 2004 to 2015
Source: BMC Ophthalmol. 2021 Nov 26;21:406. doi: 10.1186/s12886-021-02157-1 (PMC8627102; doi:10.1186/s12886-021-02157-1)
Supplement: Supplementary file 2 — Additional file 2: Supplemental Table 1. Numbers According to Age Groups and Sex in the 2004 Midyear Population in Korea. Supplemental Table 2. Numbers According to Age Groups and Sex in the 2005 Midyear Population in Korea. Supplemental Table 3. Numbers According to Age Groups and Sex in the 2006 Midyear Population in Korea. Supplemental Table 4. Numbers According to Age Groups and Sex in the 2007 Midyear Population in Korea. Supplemental Table 5. Numbers According to Age Groups and Sex in the 2008 Midyear Population in Korea. Supplemental Table 6. Numbers According to Age Groups and Sex in the 2009 Midyear Population in Korea. Supplemental Table 7. Numbers According to Age Groups and Sex in the 2010 Midyear Population in Korea. Supplemental Table 8. Numbers According to Age Groups and Sex in the 2011 Midyear Population in Korea. Supplemental Table 9. Numbers According to Age Groups and Sex in the 2012 Midyear Population in Korea. Supplemental Table 10. Numbers According to Age Groups and Sex in the 2013 Midyear Population in Korea. Supplemental Table 11. Numbers According to Age Groups and Sex in the 2014 Midyear Population in Korea. Supplemental Table 12. Numbers According to Age Groups and Sex in the 2015 Midyear Population in Korea. Supplemental Table 13. Numbers according to age groups and sex in the 2015 Census of Population in Korea. Supplemental Table 14. Number of Cases of Rhegmatogenous Retinal Detachment (RRD) Requiring Surgery and Crude and Age- and Sex-Standardized Incidence Rate of RRD in 2004. Supplemental Table 15. Number of Cases of Rhegmatogenous Retinal Detachment (RRD) Requiring Surgery and Crude and Age- and Sex-Standardized Incidence Rate of RRD in 2005. Supplemental Table 16. Number of Cases of Rhegmatogenous Retinal Detachment (RRD) Requiring Surgery and Crude and Age- and Sex-Standardized Incidence Rate of RRD in 2006. Supplemental Table 17. Number of Cases of Rhegmatogenous Retinal Detachment (RRD) Requiring Surgery and Crude and Age- and [file 12886_2021_2157_MOESM2_ESM.docx]

**Supplemental Tables**

**Supplemental Table 1. Numbers According to Age Groups and Sex in the 2004 Midyear Population in Korea**

**Supplemental Table 2. Numbers According to Age Groups and Sex in the 2005 Midyear Population in Korea**

**Supplemental Table 3. Numbers According to Age Groups and Sex in the 2006 Midyear Population in Korea**

**Supplemental Table 4. Numbers According to Age Groups and Sex in the 2007 Midyear Population in Korea**

**Supplemental Table 5. Numbers According to Age Groups and Sex in the 2008 Midyear Population in Korea**

**Supplemental Table 6. Numbers According to Age Groups and Sex in the 2009 Midyear Population in Korea**

**Supplemental Table 7. Numbers According to Age Groups and Sex in the 2010 Midyear Population in Korea**

**Supplemental Table 8. Numbers According to Age Groups and Sex in the 2011 Midyear Population in Korea**

**Supplemental Table 9. Numbers According to Age Groups and Sex in the 2012 Midyear Population in Korea**

**Supplemental Table 10. Numbers According to Age Groups and Sex in the 2013 Midyear Population in Korea**

**Supplemental Table 11. Numbers According to Age Groups and Sex in the 2014 Midyear Population in Korea**

**Supplemental Table 12. Numbers According to Age Groups and Sex in the 2015 Midyear Population in Korea**

**Supplemental Table 13. Numbers according to age groups and sex in the 2015 Census of Population in Korea**

**Supplemental Table 14. Number of Cases of Rhegmatogenous Retinal Detachment (RRD) Requiring Surgery and Crude and Age- and Sex-Standardized Incidence Rate of RRD in 2004**

**Supplemental Table 15. Number of Cases of Rhegmatogenous Retinal Detachment (RRD) Requiring Surgery and Crude and Age- and Sex-Standardized Incidence Rate of RRD in 2005**

**Supplemental Table 16. Number of Cases of Rhegmatogenous Retinal Detachment (RRD) Requiring Surgery and Crude and Age- and Sex-Standardized Incidence Rate of RRD in 2006**

**Supplemental Table 17. Number of Cases of Rhegmatogenous Retinal Detachment (RRD) Requiring Surgery and Crude and Age- and Sex-Standardized Incidence Rate of RRD in 2007**

**Supplemental Table 18. Number of Cases of Rhegmatogenous Retinal Detachment (RRD) Requiring Surgery and Crude and Age- and Sex-Standardized Incidence Rate of RRD in 2008**

**Supplemental Table 19. Number of Cases of Rhegmatogenous Retinal Detachment (RRD) Requiring Surgery and Crude and Age- and Sex-Standardized Incidence Rate of RRD in 2009**

**Supplemental Table 20. Number of Cases of Rhegmatogenous Retinal Detachment (RRD) Requiring Surgery and Crude and Age- and Sex-Standardized Incidence Rate of RRD in 2010**

**Supplemental Table 21. Number of Cases of Rhegmatogenous Retinal Detachment (RRD) Requiring Surgery and Crude and Age- and Sex-Standardized Incidence Rate of RRD in 2011**

**Supplemental Table 22. Number of Cases of Rhegmatogenous Retinal Detachment (RRD) Requiring Surgery and Crude and Age- and Sex-Standardized Incidence Rate of RRD in 2012**

**Supplemental Table 23. Number of Cases of Rhegmatogenous Retinal Detachment (RRD) Requiring Surgery and Crude and Age- and Sex-Standardized Incidence Rate of RRD in 2013**

**Supplemental Table 24. Number of Cases of Rhegmatogenous Retinal Detachment (RRD) Requiring Surgery and Crude and Age- and Sex-Standardized Incidence Rate of RRD in 2014**

**Supplemental Table 25. Number of Cases of Rhegmatogenous Retinal Detachment (RRD) Requiring Surgery and Crude and Age- and Sex-Standardized Incidence Rate of RRD in 2015**

**Supplemental Table 1. Numbers According to Age Groups and Sex in the 2004 Midyear Population in Korea**

|  | **2004 mid-year population** | | |
| --- | --- | --- | --- |
|  | **Total** | **Male** | **Female** |
| 0–4 | 2,705,352 | 1,411,847 | 1,293,505 |
| 5–9 | 3,381,814 | 1,778,589 | 1,603,225 |
| 10–14 | 3,457,590 | 1,838,148 | 1,619,442 |
| 15–19 | 3,159,628 | 1,651,535 | 1,508,093 |
| 20–24 | 3,981,199 | 2,045,753 | 1,935,446 |
| 25–29 | 3,890,299.5 | 1,986,486.5 | 1,903,813 |
| 30–34 | 4,546,133.5 | 2,319,792.5 | 2,226,341 |
| 35–39 | 4,353,161 | 2,216,884.5 | 2,136,276.5 |
| 40–44 | 4,404,869.5 | 2,249,286 | 2,155,583.5 |
| 45–49 | 3,785,353.5 | 1,921,769 | 1,863,584.5 |
| 50–54 | 2,635,665 | 1,325,468 | 1,310,197 |
| 55–59 | 2,149,594.5 | 1,062,288.5 | 1,087,306 |
| 60–64 | 2,019,689.5 | 950,918.5 | 1,068,771 |
| 65–69 | 1,621,190.5 | 718,220.5 | 902,970 |
| 70–74 | 1,085,010.5 | 421,796.5 | 663,214 |
| 75–79 | 681,460.5 | 235,148.5 | 446,312 |
| 80–84 | 399,144 | 126,506.5 | 272,637.5 |
| 85–89 | 163,755.5 | 43,225 | 120,530.5 |
| 90–94 | 52,453.5 | 11,110 | 41,343.5 |
| 95– | 11,951 | 1,840.5 | 10,110.0 |
| Total | 48,485,314 | 24,316,613 | 24,168,701 |

Available at: http://kosis.kr; accessed July 1, 2019

**Supplemental Table 2. Numbers According to Age Groups and Sex in the 2005 Midyear Population in Korea**

|  | **2005 mid-year population** | | |
| --- | --- | --- | --- |
|  | **Total** | **Male** | **Female** |
| 0–4 | 2,531,164.5 | 1,318,437.5 | 1,212,727 |
| 5–9 | 3,297,888 | 1,727,771.5 | 1,570,116.5 |
| 10–14 | 3,532,629.5 | 1,879,134.5 | 1,653,495 |
| 15–19 | 3,137,402.5 | 1,646,915.5 | 1,490,487 |
| 20–24 | 3,802,950.5 | 1,959,368.5 | 1,843,582 |
| 25–29 | 3,894,503.5 | 1,989,036 | 1,905,467.5 |
| 30–34 | 4,440,949.5 | 2,265,841 | 2,175,108.5 |
| 35–39 | 4,418,296 | 2,248,365 | 2,169,931 |
| 40–44 | 4,338,463 | 2,217,681 | 2,120,782 |
| 45–49 | 3,986,582 | 2,021,726.5 | 1,964,855.5 |
| 50–54 | 2,829,603.5 | 1,421,545 | 1,408,058.5 |
| 55–59 | 2,249,969.5 | 1,116,000 | 1,133,969.5 |
| 60–64 | 1,997,902.5 | 944,510 | 1,053,392.5 |
| 65–69 | 1,676,881 | 749,324.5 | 927,556.5 |
| 70–74 | 1,162,612 | 461,654 | 700,958 |
| 75–79 | 727,178.5 | 249,944.5 | 477,234 |
| 80–84 | 416,340 | 131,641.5 | 284,698.5 |
| 85–89 | 173,749.5 | 47,053 | 126,696.5 |
| 90–94 | 55,065 | 11,497 | 43,568 |
| 95– | 12,909 | 2,212.0 | 10,697.0 |
| Total | 48,683,039.5 | 24,409,658.5 | 24,273,381 |

Available at: http://kosis.kr; accessed July 1, 2019

**Supplemental Table 3. Numbers According to Age Groups and Sex in the 2006 Midyear Population in Korea**

|  | **2006 mid-year population** | | |
| --- | --- | --- | --- |
|  | **Total** | **Male** | **Female** |
| 0–4 | 2,373,071 | 1,233,701 | 1,139,370 |
| 5–9 | 3,198,104 | 1,671,001.5 | 1,527,102.5 |
| 10–14 | 3,557,222 | 1,890,244 | 1,666,978 |
| 15–19 | 3,168,789.5 | 1,668,896.5 | 1,499,893 |
| 20–24 | 3,601,632 | 1,860,647 | 1,740,985 |
| 25–29 | 3,955,326 | 2,022,829.5 | 1,932,496.5 |
| 30–34 | 4,283,008.5 | 2,183,179.5 | 2,099,829 |
| 35–39 | 4,506,625 | 2,289,147 | 2,217,478 |
| 40–44 | 4,237,804 | 2,174,572.5 | 2,063,231.5 |
| 45–49 | 4,180,169 | 2,118,789 | 2,061,380 |
| 50–54 | 3,084,155.5 | 1,547,434.5 | 1,536,721 |
| 55–59 | 2,327,211 | 1,155,201.5 | 1,172,009.5 |
| 60–64 | 1,973,280.5 | 939,135.5 | 1,034,145 |
| 65–69 | 1,728,713.5 | 778,285 | 950,428.5 |
| 70–74 | 1,249,692 | 506,635 | 743,057 |
| 75–79 | 770,065.5 | 265,102.5 | 504,963 |
| 80–84 | 433,677 | 136,109 | 297,568 |
| 85–89 | 186,086.5 | 51,044.5 | 135,042 |
| 90–94 | 58,450.5 | 12,174.5 | 46,276 |
| 95– | 13,943.5 | 2,489.5 | 11,454 |
| Total | 48,887,026.5 | 24,506,619 | 24,380,407.5 |

Available at: http://kosis.kr; accessed July 1, 2019

**Supplemental Table 4. Numbers According to Age Groups and Sex in the 2007 Midyear Population in Korea**

|  | **2007 mid-year population** | | |
| --- | --- | --- | --- |
|  | **Total** | **Male** | **Female** |
| 0–4 | 2,316,154.5 | 1,201,209 | 1,114,945.5 |
| 5–9 | 3,044,538.5 | 1,589,915 | 1,454,623.5 |
| 10–14 | 3,521,067 | 1,867,106 | 1,653,961 |
| 15–19 | 3,261,491.5 | 1,722,228.5 | 1,539,263 |
| 20–24 | 3,390,578 | 1,756,562 | 1,634,016 |
| 25–29 | 4,014,120.5 | 2,056,125 | 1,957,995.5 |
| 30–34 | 4,133,156.5 | 2,102,838 | 2,030,318.5 |
| 35–39 | 4,581,291 | 2,328,233 | 2,253,058 |
| 40–44 | 4,168,839.5 | 2,139,080 | 2,029,759.5 |
| 45–49 | 4,329,573.5 | 2,194,685.5 | 2,134,888 |
| 50–54 | 3,299,005 | 1,658,052 | 1,640,953 |
| 55–59 | 2,402,705 | 1,193,630 | 1,209,075 |
| 60–64 | 1,958,728.5 | 938,689 | 1,020,039.5 |
| 65–69 | 1,830,670 | 830,647.5 | 1,000,022.5 |
| 70–74 | 1,328,156 | 547,610.5 | 780,545.5 |
| 75–79 | 817,037 | 284,446 | 532,591 |
| 80–84 | 452,232.5 | 141,170.5 | 311,062 |
| 85–89 | 202,834 | 55,978.5 | 146,855.5 |
| 90–94 | 62,984.5 | 13,223.5 | 49,761 |
| 95– | 15,191 | 2,697 | 12,494 |
| Total | 49,130,353.5 | 24,624,126.5 | 24,506,227 |

Available at: http://kosis.kr; accessed July 1, 2019

**Supplemental Table 5. Numbers According to Age Groups and Sex in the 2008 Midyear Population in Korea**

|  | **2008 mid-year population** | | |
| --- | --- | --- | --- |
|  | **Total** | **Male** | **Female** |
| 0–4 | 2,302,912 | 1,191,064.5 | 1,111,847.5 |
| 5–9 | 2,880,926.5 | 1,504,441.5 | 1,376,485 |
| 10–14 | 3,457,992.5 | 1,826,142 | 1,631,850.5 |
| 15–19 | 3,359,504 | 1,779,842.5 | 1,579,661.5 |
| 20–24 | 3,225,672.5 | 1,676,808 | 1,548,864.5 |
| 25–29 | 4,048,159 | 2,074,424 | 1,973,735 |
| 30–34 | 3,989,906.5 | 2,031,664.5 | 1,958,242 |
| 35–39 | 4,580,942.5 | 2,334,773.5 | 2,246,169 |
| 40–44 | 4,222,513 | 2,153,919 | 2,068,594 |
| 45–49 | 4,380,451.5 | 2,223,951 | 2,156,500.5 |
| 50–54 | 3,505,440 | 1,766,719.5 | 1,738,720.5 |
| 55–59 | 2,486,210 | 1,235,411.5 | 1,250,798.5 |
| 60–64 | 1,998,643 | 965,080.5 | 1,033,562.5 |
| 65–69 | 1,911,106.5 | 872,145 | 1,038,961.5 |
| 70–74 | 1,401,770.5 | 586,226 | 815,544.5 |
| 75–79 | 867,555 | 307,567.5 | 559,987.5 |
| 80–84 | 476,336.5 | 147,805.5 | 328,531 |
| 85–89 | 224,863.5 | 61,854 | 163,009.5 |
| 90–94 | 67,252 | 14,326.5 | 52,925.5 |
| 95– | 16,491 | 2,907 | 13,584 |
| Total | 49,404,647.5 | 24,757,073 | 24,647,574.5 |

Available at: http://kosis.kr; accessed July 1, 2019**Supplemental Table 6. Numbers According to Age Groups and Sex in the 2009 Midyear Population in Korea**

|  | **2009 mid-year population** | | |
| --- | --- | --- | --- |
|  | **Total** | **Male** | **Female** |
| 0–4 | 2,276,741.5 | 1,175,453 | 1,101,288.5 |
| 5–9 | 2,733,174.5 | 1,425,237 | 1,307,937.5 |
| 10–14 | 3,374,071 | 1,773,968.5 | 1,600,102.5 |
| 15–19 | 3,444,215 | 1,829,732.5 | 1,614,482.5 |
| 20–24 | 3,149,080.5 | 1,643,101 | 1,505,979.5 |
| 25–29 | 3,977,869.5 | 2,039,125.5 | 1,938,744 |
| 30–34 | 3,888,698.5 | 1,983,235.5 | 1,905,463 |
| 35–39 | 4,532,215 | 2,311,986.5 | 2,220,228.5 |
| 40–44 | 4,331,690 | 2,202,463.5 | 2,129,226.5 |
| 45–49 | 4,368,981.5 | 2,222,229 | 2,146,752.5 |
| 50–54 | 3,738,881.5 | 1,884,660 | 1,854,221.5 |
| 55–59 | 2,585,443.5 | 1,285,563.5 | 1,299,880 |
| 60–64 | 2,087,203.5 | 1,015,578.5 | 1,071,625 |
| 65–69 | 1,918,940.5 | 879,148.5 | 1,039,792 |
| 70–74 | 1,479,452.5 | 626,301 | 853,151.5 |
| 75–79 | 925,787 | 334,940.5 | 590,846.5 |
| 80–84 | 511,783 | 158,094 | 353,689 |
| 85–89 | 242,801.5 | 66,732 | 176,069.5 |
| 90–94 | 71,894 | 15,787.5 | 56,106.5 |
| 95– | 17,832 | 3,080.5 | 14,751.5 |
| Total | 49,656,756 | 24,876,418 | 24,780,338 |

Available at: http://kosis.kr; accessed July 1, 2019

**Supplemental Table 7. Numbers According to Age Groups and Sex in the 2010 Midyear Population in Korea**

|  | **2010 mid-year population** | | |
| --- | --- | --- | --- |
|  | **Total** | **Male** | **Female** |
| 0–4 | 2,281,200 | 1,176,430 | 1,104,770 |
| 5–9 | 2,557,314.5 | 1,331,287 | 1,226,027.5 |
| 10–14 | 3,290,667.5 | 1,723,436.5 | 1,567,231 |
| 15–19 | 3,519,339.5 | 1,870,870 | 1,648,469.5 |
| 20–24 | 3,127,804 | 1,638,804 | 1,489,000 |
| 25–29 | 3,799,704.5 | 1,951,930 | 1,847,774.5 |
| 30–34 | 3,893,137.5 | 1,984,565.5 | 1,908,572 |
| 35–39 | 4,431,641.5 | 2,260,208 | 2,171,433.5 |
| 40–44 | 4,403,553.5 | 2,237,800 | 2,165,753.5 |
| 45–49 | 4,312,085 | 2,196,291 | 2,115,794 |
| 50–54 | 3,945,392 | 1,987,063 | 1,958,329 |
| 55–59 | 2,781,323.5 | 1,382,357 | 1,398,966.5 |
| 60–64 | 2,188,466.5 | 1,069,407 | 1,119,059.5 |
| 65–69 | 1,904,258 | 877,420.5 | 1,026,837.5 |
| 70–74 | 1,537,053.5 | 657,441.5 | 879,612 |
| 75–79 | 999,588.5 | 370,465.5 | 629,123 |
| 80–84 | 553,441 | 170,484.5 | 382,956.5 |
| 85–89 | 256,657.5 | 70,007.5 | 186,650 |
| 90–94 | 78,042.5 | 17,641.5 | 60,401 |
| 95– | 19,141 | 3,253.5 | 15,887.5 |
| Total | 49,879,811.5 | 24,977,163.5 | 24,902,648 |

Available at: http://kosis.kr; accessed July 1, 2019

**Supplemental Table 8. Numbers According to Age Groups and Sex in the 2011 Midyear Population in Korea**

|  | **2011 mid-year population** | | |
| --- | --- | --- | --- |
|  | **Total** | **Male** | **Female** |
| 0–4 | 2,313,461 | 1,191,773 | 1,121,688 |
| 5–9 | 2,400,178.5 | 1,247,218 | 1,152,960.5 |
| 10–14 | 3,191,849 | 1,667,248 | 1,524,601 |
| 15–19 | 3,544,677 | 1,882,478 | 1,662,199 |
| 20–24 | 3,160,370.5 | 1,661,130 | 1,499,240.5 |
| 25–29 | 3,599,264.5 | 1,852,499.5 | 1,746,765 |
| 30–34 | 3,952,330.5 | 2,016,304.5 | 1,936,026 |
| 35–39 | 4,273,860.5 | 2,176,905.5 | 2,096,955 |
| 40–44 | 4,492,969 | 2,278,987 | 2,213,982 |
| 45–49 | 4,216,075.5 | 2,156,068 | 2,060,007.5 |
| 50–54 | 4,140,623 | 2,084,985.5 | 2,055,637.5 |
| 55–59 | 3,035,090 | 1,507,151 | 1,527,939 |
| 60–64 | 2,265,097 | 1,107,881.5 | 1,157,215.5 |
| 65–69 | 1,885,440.5 | 875,945.5 | 1,009,495 |
| 70–74 | 1,591,015.5 | 686,847.5 | 904,168 |
| 75–79 | 1,080,423.5 | 409,659 | 670,764.5 |
| 80–84 | 592,117.5 | 182,886.5 | 409,231 |
| 85–89 | 270,553.5 | 72,911 | 197,642.5 |
| 90–94 | 85,407.5 | 19,488 | 65,919.5 |
| 95– | 20,672 | 3,421 | 17,251 |
| Total | 50,111,475.5 | 25,081,787.5 | 25,029,688 |

Available at: http://kosis.kr; accessed July 1, 2019

**Supplemental Table 9. Numbers According to Age Groups and Sex in the 2012 Midyear Population in Korea**

|  | **2012 mid-year population** | | |
| --- | --- | --- | --- |
|  | **Total** | **Male** | **Female** |
| 0–4 | 2,324,347 | 1,196,279.5 | 1,128,067.5 |
| 5–9 | 2,345,370.5 | 1,215,826 | 1,129,544.5 |
| 10–14 | 3,039,416 | 1,586,749 | 1,452,667 |
| 15–19 | 3,508,863.5 | 1,859,556 | 1,649,307.5 |
| 20–24 | 3,252,668.5 | 1,713,945.5 | 1,538,723 |
| 25–29 | 3,390,402.5 | 1,748,146 | 1,642,256.5 |
| 30–34 | 4,010,160 | 2,047,847.5 | 1,962,312.5 |
| 35–39 | 4,122,957.5 | 2,095,268.5 | 2,027,689 |
| 40–44 | 4,565,850 | 2,316,941 | 2,248,909 |
| 45–49 | 4,147,568.5 | 2,120,985.5 | 2,026,583 |
| 50–54 | 4,289,309 | 2,160,551 | 2,128,758 |
| 55–59 | 3,247,999.5 | 1,616,101.5 | 1,631,898 |
| 60–64 | 2,340,619 | 1,145,902.5 | 1,194,716.5 |
| 65–69 | 1,877,115.5 | 879,626 | 997,489.5 |
| 70–74 | 1,692,241 | 737,953 | 954,288 |
| 75–79 | 1,153,246 | 445,317 | 707,929 |
| 80–84 | 633,683.5 | 198,522 | 435,161.5 |
| 85–89 | 285,890.5 | 76,553 | 209,337.5 |
| 90–94 | 95,032 | 21,711 | 73,321 |
| 95– | 22,585 | 3,713 | 18,872 |
| Total | 50,345,324.5 | 25,187,494 | 25,157,830.5 |

Available at: http://kosis.kr; accessed July 1, 2019

**Supplemental Table 10. Numbers According to Age Groups and Sex in the 2013 Midyear Population in Korea**

|  | **2013 mid-year population** | | |
| --- | --- | --- | --- |
|  | **Total** | **Male** | **Female** |
| 0–4 | 2,310,730.5 | 1,188,546.5 | 1,122,184 |
| 5–9 | 2,333,194 | 1,206,347 | 1,126,847 |
| 10–14 | 2,877,382 | 1,502,120 | 1,375,262 |
| 15–19 | 3,446,369.5 | 1,818,948.5 | 1,627,421 |
| 20–24 | 3,349,079 | 1,770,703.5 | 1,578,375.5 |
| 25–29 | 3,226,780.5 | 1,667,899.5 | 1,558,881 |
| 30–34 | 4,042,034 | 2,063,995 | 1,978,039 |
| 35–39 | 3,976,799 | 2,021,618 | 1,955,181 |
| 40–44 | 4,560,614 | 2,320,135.5 | 2,240,478.5 |
| 45–49 | 4,195,939 | 2,132,558 | 2,063,381 |
| 50–54 | 4,336,465 | 2,187,739.5 | 2,148,725.5 |
| 55–59 | 3,449,858 | 1,721,494.5 | 1,728,363.5 |
| 60–64 | 2,423,152 | 1,186,814 | 1,236,338 |
| 65–69 | 1,920,233 | 908,015 | 1,012,218 |
| 70–74 | 1,772,314 | 779,034 | 993,280 |
| 75–79 | 1,222,240 | 479,575 | 742,665 |
| 80–84 | 678,338 | 217,303 | 461,035 |
| 85–89 | 305,839.5 | 81,430.5 | 224,409 |
| 90–94 | 107,274 | 24,581.5 | 82,692.5 |
| 95– | 24,316.5 | 4,069.5 | 20,247 |
| Total | 50,558,951.5 | 25,282,928 | 25,276,023.5 |

Available at: http://kosis.kr; accessed July 1, 2019

**Supplemental Table 11. Numbers According to Age Groups and Sex in the 2014 Midyear Population in Korea**

|  | **2014 mid-year population** | | |
| --- | --- | --- | --- |
|  | **Total** | **Male** | **Female** |
| 0–4 | 2,297,243.5 | 1,180,475.5 | 1,116,768 |
| 5–9 | 2,308,229 | 1,191,389.5 | 1,116,839.5 |
| 10–14 | 2,731,443 | 1,423,962.5 | 1,307,480.5 |
| 15–19 | 3,364,378.5 | 1,767,803 | 1,596,575.5 |
| 20–24 | 3,433,785.5 | 1,820,690.5 | 1,613,095 |
| 25–29 | 3,151,400.5 | 1,634,327 | 1,517,073.5 |
| 30–34 | 3,971,975.5 | 2,028,612 | 1,943,363.5 |
| 35–39 | 3,875,142.5 | 1,972,312.5 | 1,902,830 |
| 40–44 | 4,509,393.5 | 2,295,490.5 | 2,213,903 |
| 45–49 | 4,301,489.5 | 2,178,993.5 | 2,122,496 |
| 50–54 | 4,322,181.5 | 2,184,689 | 2,137,492.5 |
| 55–59 | 3,678,401.5 | 1,836,247.5 | 1,842,154 |
| 60–64 | 2,521,163 | 1,236,224.5 | 1,284,938.5 |
| 65–69 | 2,008,782 | 958,043.5 | 1,050,738.5 |
| 70–74 | 1,785,086.5 | 789,396.5 | 995,690 |
| 75–79 | 1,296,333.5 | 516,035 | 780,298.5 |
| 80–84 | 729,676 | 239,458 | 490,218 |
| 85–89 | 333,691.5 | 88,734.5 | 244,957 |
| 90–94 | 117,059.5 | 27,027.5 | 90,032 |
| 95– | 26,302 | 4,574 | 21,729 |
| Total | 50,763,158 | 25,374,486 | 25,388,672 |

Available at: http://kosis.kr; accessed July 1, 2019

**Supplemental Table 12. Numbers According to Age Groups and Sex in the 2015 Midyear Population in Korea**

|  | **2015 mid-year population** | | |
| --- | --- | --- | --- |
|  | **Total** | **Male** | **Female** |
| 0–4 | 2,279,974.5 | 1,170,260.5 | 1,109,714 |
| 5–9 | 2,314,310.5 | 1,193,117.5 | 1,121,193 |
| 10–14 | 2,557,069 | 1,330,833.5 | 1,226,235.5 |
| 15–19 | 3,283,016 | 1,718,355 | 1,564,661 |
| 20–24 | 3,509,440 | 1,862,369.5 | 1,647,070.5 |
| 25–29 | 3,130,239 | 1,630,423.5 | 1,499,815.5 |
| 30–34 | 3,796,511 | 1,942,875 | 1,853,636 |
| 35–39 | 3,881,395.5 | 1,974,398.5 | 1,906,997 |
| 40–44 | 4,410,395.5 | 2,244,823 | 2,165,572.5 |
| 45–49 | 4,372,918 | 2,214,502 | 2,158,416 |
| 50–54 | 4,266,240.5 | 2,159,896 | 2,106,344.5 |
| 55–59 | 3,882,543 | 1,937,358.5 | 1,945,184.5 |
| 60–64 | 2,715,138.5 | 1,331,686.5 | 1,383,452 |
| 65–69 | 2,109,479 | 1,010,889 | 1,098,590 |
| 70–74 | 1,777,348 | 791,986 | 985,362 |
| 75–79 | 1,353,621.5 | 545,835 | 807,786.5 |
| 80–84 | 794,235.5 | 267,805.5 | 526,430 |
| 85–89 | 365,080 | 97,021 | 268,059 |
| 90–94 | 123,946 | 28,401 | 95,545 |
| 95– | 28,818 | 5,221.0 | 23,597.0 |
| Total | 50,951,719 | 25,458,057.5 | 25,493,661.5 |

Available at: http://kosis.kr; accessed July 1, 2019

**Supplemental Table 13. Numbers according to age groups and sex in the 2015 Census of Population in Korea**

|  | **2015 Census population** | | |
| --- | --- | --- | --- |
|  | **Total** | **Male** | **Female** |
| 0–4 | 2,235,397 | 1,147,126 | 1,088,271 |
| 5–9 | 2,252,950 | 1,162,087 | 1,090,863 |
| 10–14 | 2,418,360 | 1,257,902 | 1,160,458 |
| 15–19 | 3,170,545 | 1,657,722 | 1,512,823 |
| 20–24 | 3,385,936 | 1,808,857 | 1,577,079 |
| 25–29 | 3,027,896 | 1,581,887 | 1,446,009 |
| 30–34 | 3,611,034 | 1,854,905 | 1,756,129 |
| 35–39 | 3,783,589 | 1,927,388 | 1,856,201 |
| 40–44 | 4,215,921 | 2,142,101 | 2,073,820 |
| 45–49 | 4,266,941 | 2,151,070 | 2,115,871 |
| 50–54 | 4,145,976 | 2,094,318 | 2,051,658 |
| 55–59 | 3,863,095 | 1,922,796 | 1,940,299 |
| 60–64 | 2,758,941 | 1,348,273 | 1,410,668 |
| 65–69 | 2,117,875 | 1,015,463 | 1,102,412 |
| 70–74 | 1,760,932 | 789,607 | 971,325 |
| 75–79 | 1,356,014 | 550,684 | 805,330 |
| 80–84 | 810,891 | 275,462 | 535,429 |
| 85–89 | 371,527 | 98,367 | 273,160 |
| 90–94 | 124,111 | 28,565 | 95,546 |
| 95– | 27,732 | 5259 | 22,473 |
| Total | 49,705,663 | 24,819,839 | 24,885,824 |

Available at: http://kosis.kr; accessed July 1, 2019

**Supplemental Table 14. Number of Cases of Rhegmatogenous Retinal Detachment (RRD) Requiring Surgery and Crude and Age- and Sex-Standardized Incidence Rate of RRD in 2004**

|  | **N of Cases** | | | **Crude Incidence Rate (per 1,000,000 person-years)** | | | | | |  |
| --- | --- | --- | --- | --- | --- | --- | --- | --- | --- | --- |
| **Age** | **Total** | **Male** | **Female** | **Total** | **95%CI** | **Male** | **95%CI** | **Female** | **95%CI** | **M:F Ratio** |
| 0–4 | NA | NA | NA | NA | NA | NA | NA | NA | NA |  |
| 5–9 | 5 | NA | NA | 0.15 | (0.02–0.28) | NA | NA | NA | NA |  |
| 10–14 | 47 | 36 | 11 | 1.36 | (0.97–1.75) | 1.96 | (1.32–2.60) | 0.68 | (0.28–1.08) | 2.88 |
| 15–19 | 139 | 104 | 35 | 4.40 | (3.67–5.13) | 6.30 | (5.09–7.51) | 2.32 | (1.55–3.09) | 2.71 |
| 20–24 | 211 | 111 | 100 | 5.30 | (4.58–6.02) | 5.43 | (4.42–6.44) | 5.17 | (4.15–6.18) | 1.05 |
| 25–29 | 196 | 104 | 92 | 5.04 | (4.33–5.74) | 5.24 | (4.23–6.24) | 4.83 | (3.84–5.82) | 1.08 |
| 30–34 | 171 | 103 | 68 | 3.76 | (3.20–4.33) | 4.44 | (3.58–5.30) | 3.05 | (2.33–3.78) | 1.45 |
| 35–39 | 179 | 128 | 51 | 4.11 | (3.51–4.71) | 5.77 | (4.77–6.77) | 2.39 | (1.73–3.04) | 2.42 |
| 40–44 | 209 | 121 | 88 | 4.74 | (4.10–5.39) | 5.38 | (4.42–6.34) | 4.08 | (3.23–4.94) | 1.32 |
| 45–49 | 255 | 165 | 90 | 6.74 | (5.91–7.56) | 8.59 | (7.28–9.90) | 4.83 | (3.83–5.83) | 1.78 |
| 50–54 | 291 | 159 | 132 | 11.04 | (9.77–12.31) | 12.00 | (10.13–13.86) | 10.07 | (8.36–11.79) | 1.19 |
| 55–59 | 329 | 163 | 166 | 15.31 | (13.65–16.96) | 15.34 | (12.99–17.70) | 15.27 | (12.94–17.59) | 1.01 |
| 60–64 | 386 | 184 | 202 | 19.11 | (17.21–21.02) | 19.35 | (16.55–22.15) | 18.90 | (16.29–21.51) | 1.02 |
| 65–69 | 292 | 139 | 153 | 18.01 | (15.95–20.08) | 19.35 | (16.14–22.57) | 16.94 | (14.26–19.63) | 1.14 |
| 70–74 | 147 | 65 | 82 | 13.55 | (11.36–15.74) | 15.41 | (11.66–19.16) | 12.36 | (9.69–15.04) | 1.25 |
| 75–79 | 70 | 32 | 38 | 10.27 | (7.87–12.68) | 13.61 | (8.89–18.32) | 8.51 | (5.81–11.22) | 1.60 |
| 80–84 | 14 | 7 | 7 | 3.51 | (1.67–5.34) | 5.53 | (1.43–9.63) | 2.57 | (0.67–4.47) | 2.16 |
| 85–89 | NA | NA | NA | NA | NA | NA | NA | NA | NA |  |
| 90–94 | NA | NA | NA | NA | NA | NA | NA | NA | NA |  |
| 95– | NA | NA | NA | NA | NA | NA | NA | NA | NA |  |
| Total | 2947 | 1627 | 1320 | 6.08 | (5.86–6.30) | 6.69 | (6.37–7.02) | 5.46 | (5.17–5.76) | 1.23 |
|  |  |  |  |  |  |  |  |  |  |  |
|  | **Standardized N of Cases** | | | **Standardized Incidence Rate (per 1,000,000 person-years)*** | | | | | |  |
|  | **Total** | **Male** | **Female** | **Total** | **95%CI** | **Male** | **95%CI** | **Female** | **95%CI** | **M:F Ratio** |
| Total | 3661.6 | 2028.2 | 1643.3 | 7.39 | (7.15–7.63) | 8.17 | (7.82–8.53) | 6.60 | (6.28–6.92) | 1.24 |

***In reference to the 2015 Census Data**

**NA; not availableSupplemental Table 15. Number of Cases of Rhegmatogenous Retinal Detachment (RRD) Requiring Surgery and Crude and Age- and Sex-Standardized Incidence Rate of RRD in 2005**

|  | **N of Cases** | | | **Crude Incidence Rate (per 1,000,000 person-years)** | | | | | |  |
| --- | --- | --- | --- | --- | --- | --- | --- | --- | --- | --- |
| **Age** | **Total** | **Male** | **Female** | **Total** | **95%CI** | **Male** | **95%CI** | **Female** | **95%CI** | **M:F Ratio** |
| 0–4 | NA | NA | NA | NA | NA | NA | NA | NA | NA |  |
| 5–9 | 8 | 6 | NA | 0.24 | (0.07–0.41) | 0.35 | (0.07–0.63) | NA | NA |  |
| 10–14 | 56 | 45 | 11 | 1.59 | (1.17–2.00) | 2.39 | (1.70–3.09) | 0.67 | (0.27–1.06) | 3.60 |
| 15–19 | 157 | 108 | 49 | 5.00 | (4.22–5.79) | 6.56 | (5.32–7.79) | 3.29 | (2.37–4.21) | 1.99 |
| 20–24 | 245 | 115 | 130 | 6.44 | (5.64–7.25) | 5.87 | (4.80–6.94) | 7.05 | (5.84–8.26) | 0.83 |
| 25–29 | 206 | 102 | 104 | 5.29 | (4.57–6.01) | 5.13 | (4.13–6.12) | 5.46 | (4.41–6.51) | 0.94 |
| 30–34 | 206 | 112 | 94 | 4.64 | (4.01–5.27) | 4.94 | (4.03–5.86) | 4.32 | (3.45–5.20) | 1.14 |
| 35–39 | 179 | 115 | 64 | 4.05 | (3.46–4.64) | 5.11 | (4.18–6.05) | 2.95 | (2.23–3.67) | 1.73 |
| 40–44 | 233 | 144 | 89 | 5.37 | (4.68–6.06) | 6.49 | (5.43–7.55) | 4.20 | (3.32–5.07) | 1.55 |
| 45–49 | 353 | 214 | 139 | 8.85 | (7.93–9.78) | 10.59 | (9.17–12.00) | 7.07 | (5.90–8.25) | 1.50 |
| 50–54 | 372 | 215 | 157 | 13.15 | (11.81–14.48) | 15.12 | (13.10–17.15) | 11.15 | (9.41–12.89) | 1.36 |
| 55–59 | 427 | 218 | 209 | 18.98 | (17.18–20.78) | 19.53 | (16.94–22.13) | 18.43 | (15.93–20.93) | 1.06 |
| 60–64 | 419 | 209 | 210 | 20.97 | (18.96–22.98) | 22.13 | (19.13–25.13) | 19.94 | (17.24–22.63) | 1.11 |
| 65–69 | 347 | 161 | 186 | 20.69 | (18.52–22.87) | 21.49 | (18.17–24.80) | 20.05 | (17.17–22.93) | 1.07 |
| 70–74 | 187 | 88 | 99 | 16.08 | (13.78–18.39) | 19.06 | (15.08–23.04) | 14.12 | (11.34–16.91) | 1.35 |
| 75–79 | 67 | 30 | 37 | 9.21 | (7.01–11.42) | 12.00 | (7.71–16.30) | 7.75 | (5.25–10.25) | 1.55 |
| 80–84 | 17 | 6 | 11 | 4.08 | (2.14–6.02) | 4.56 | (0.91–8.20) | 3.86 | (1.58–6.15) | 1.18 |
| 85–89 | 5 | NA | NA | 2.88 | (0.36–5.40) | NA | NA | NA | NA |  |
| 90–94 | NA | NA | NA | NA | NA | NA | NA | NA | NA |  |
| 95– | NA | NA | NA | NA | NA | NA | NA | NA | NA |  |
| Total | 3487 | 1892 | 1595 | 7.16 | (6.92–7.40) | 7.75 | (7.40–8.10) | 6.57 | (6.25–6.89) | 1.18 |
|  |  |  |  |  |  |  |  |  |  |  |
|  | **Standardized N of Cases** | | | **Standardized Incidence Rate (per 1,000,000 person-years)*** | | | | | |  |
|  | **Total** | **Male** | **Female** | **Total** | **95%CI** | **Male** | **95%CI** | **Female** | **95%CI** | **M:F Ratio** |
| Total | 4256.8 | 2331.2 | 1934.0 | 8.58 | (8.32–8.84) | 9.39 | (9.01–9.77) | 7.77 | (7.42–8.12) | 1.21 |

***In reference to the 2015 Census Data**

**NA; not available**

**Supplemental Table 16. Number of Cases of Rhegmatogenous Retinal Detachment (RRD) Requiring Surgery and Crude and Age- and Sex-Standardized Incidence Rate of RRD in 2006**

|  | **N of Cases** | | | **Crude Incidence Rate (per 1,000,000 person-years)** | | | | | |  |
| --- | --- | --- | --- | --- | --- | --- | --- | --- | --- | --- |
| **Age** | **Total** | **Male** | **Female** | **Total** | **95%CI** | **Male** | **95%CI** | **Female** | **95%CI** | **M:F Ratio** |
| 0–4 | NA | NA | NA | NA | NA | NA | NA | NA | NA |  |
| 5–9 | 16 | 9 | 7 | 0.50 | (0.26–0.75) | 0.54 | (0.19–0.89) | 0.46 | (0.12–0.80) | 1.17 |
| 10–14 | 71 | 59 | 12 | 2.00 | (1.53–2.46) | 3.12 | (2.32–3.92) | 0.72 | (0.31–1.13) | 4.34 |
| 15–19 | 197 | 149 | 48 | 6.22 | (5.35–7.09) | 8.93 | (7.49–10.36) | 3.20 | (2.29–4.11) | 2.79 |
| 20–24 | 246 | 128 | 118 | 6.83 | (5.98–7.68) | 6.88 | (5.69–8.07) | 6.78 | (5.55–8.00) | 1.01 |
| 25–29 | 236 | 115 | 121 | 5.97 | (5.21–6.73) | 5.69 | (4.65–6.72) | 6.26 | (5.15–7.38) | 0.91 |
| 30–34 | 197 | 113 | 84 | 4.60 | (3.96–5.24) | 5.18 | (4.22–6.13) | 4.00 | (3.14–4.86) | 1.29 |
| 35–39 | 228 | 137 | 91 | 5.06 | (4.40–5.72) | 5.98 | (4.98–6.99) | 4.10 | (3.26–4.95) | 1.46 |
| 40–44 | 226 | 137 | 89 | 5.33 | (4.64–6.03) | 6.30 | (5.25–7.36) | 4.31 | (3.42–5.21) | 1.46 |
| 45–49 | 376 | 243 | 133 | 8.99 | (8.09–9.90) | 11.47 | (10.03–12.91) | 6.45 | (5.36–7.55) | 1.78 |
| 50–54 | 403 | 215 | 188 | 13.07 | (11.79–14.34) | 13.89 | (12.04–15.75) | 12.23 | (10.49–13.98) | 1.14 |
| 55–59 | 435 | 229 | 206 | 18.69 | (16.94–20.45) | 19.82 | (17.26–22.39) | 17.58 | (15.18–19.98) | 1.13 |
| 60–64 | 449 | 206 | 243 | 22.75 | (20.65–24.86) | 21.94 | (18.94–24.93) | 23.50 | (20.54–26.45) | 0.93 |
| 65–69 | 407 | 192 | 215 | 23.54 | (21.26–25.83) | 24.67 | (21.18–28.16) | 22.62 | (19.60–25.65) | 1.09 |
| 70–74 | 235 | 87 | 148 | 18.80 | (16.40–21.21) | 17.17 | (13.56–20.78) | 19.92 | (16.71–23.13) | 0.86 |
| 75–79 | 108 | 39 | 69 | 14.02 | (11.38–16.67) | 14.71 | (10.09–19.33) | 13.66 | (10.44–16.89) | 1.08 |
| 80–84 | 37 | 13 | 24 | 8.53 | (5.78–11.28) | 9.55 | (4.36–14.74) | 8.07 | (4.84–11.29) | 1.18 |
| 85–89 | 7 | NA | NA | 3.76 | (0.97–6.55) | NA | NA | NA | NA |  |
| 90–94 | NA | NA | NA | NA | NA | NA | NA | NA | NA |  |
| 95– | NA | NA | NA | NA | NA | NA | NA | NA | NA |  |
| Total | 3875 | 2075 | 1800 | 7.93 | (7.68–8.18) | 8.47 | (8.10–8.83) | 7.38 | (7.04–7.72) | 1.15 |
|  |  |  |  |  |  |  |  |  |  |  |
|  | **Standardized N of Cases** | | | **Standardized Incidence Rate (per 1,000,000 person-years)*** | | | | | |  |
|  | **Total** | **Male** | **Female** | **Total** | **95%CI** | **Male** | **95%CI** | **Female** | **95%CI** | **M:F Ratio** |
| Total | 4631.6 | 2469.9 | 2159.3 | 9.31 | (9.05–9.58) | 9.95 | (9.56–10.34) | 8.68 | (8.31–9.04) | 1.15 |

***In reference to the 2015 Census Data**

**NA; not available**

**Supplemental Table 17. Number of Cases of Rhegmatogenous Retinal Detachment (RRD) Requiring Surgery and Crude and Age- and Sex-Standardized Incidence Rate of RRD in 2007**

|  | **N of Cases** | | | **Crude Incidence Rate (per 1,000,000 person-years)** | | | | | |  |
| --- | --- | --- | --- | --- | --- | --- | --- | --- | --- | --- |
| **Age** | **Total** | **Male** | **Female** | **Total** | **95%CI** | **Male** | **95%CI** | **Female** | **95%CI** | **M:F Ratio** |
| 0–4 | NA | NA | NA | NA | NA | NA | NA | NA | NA | 0.00 |
| 5–9 | 11 | 9 | NA | 0.36 | (0.15–0.57) | 0.57 | (0.20–0.94) | NA | NA | 4.12 |
| 10–14 | 64 | 50 | 14 | 1.82 | (1.37–2.26) | 2.68 | (1.94–3.42) | 0.85 | (0.40–1.29) | 3.16 |
| 15–19 | 177 | 127 | 50 | 5.43 | (4.63–6.23) | 7.37 | (6.09–8.66) | 3.25 | (2.35–4.15) | 2.27 |
| 20–24 | 256 | 126 | 130 | 7.55 | (6.63–8.48) | 7.17 | (5.92–8.43) | 7.96 | (6.59–9.32) | 0.90 |
| 25–29 | 275 | 139 | 136 | 6.85 | (6.04–7.66) | 6.76 | (5.64–7.88) | 6.95 | (5.78–8.11) | 0.97 |
| 30–34 | 205 | 115 | 90 | 4.96 | (4.28–5.64) | 5.47 | (4.47–6.47) | 4.43 | (3.52–5.35) | 1.23 |
| 35–39 | 247 | 168 | 79 | 5.39 | (4.72–6.06) | 7.22 | (6.12–8.31) | 3.51 | (2.73–4.28) | 2.06 |
| 40–44 | 244 | 156 | 88 | 5.85 | (5.12–6.59) | 7.29 | (6.15–8.44) | 4.34 | (3.43–5.24) | 1.68 |
| 45–49 | 382 | 229 | 153 | 8.82 | (7.94–9.71) | 10.43 | (9.08–11.79) | 7.17 | (6.03–8.30) | 1.46 |
| 50–54 | 441 | 253 | 188 | 13.37 | (12.12–14.62) | 15.26 | (13.38–17.14) | 11.46 | (9.82–13.09) | 1.33 |
| 55–59 | 535 | 285 | 250 | 22.27 | (20.38–24.15) | 23.88 | (21.10–26.65) | 20.68 | (18.11–23.24) | 1.15 |
| 60–64 | 450 | 209 | 241 | 22.97 | (20.85–25.10) | 22.27 | (19.25–25.28) | 23.63 | (20.64–26.61) | 0.94 |
| 65–69 | 424 | 203 | 221 | 23.16 | (20.96–25.37) | 24.44 | (21.08–27.80) | 22.10 | (19.19–25.01) | 1.11 |
| 70–74 | 276 | 128 | 148 | 20.78 | (18.33–23.23) | 23.37 | (19.32–27.42) | 18.96 | (15.91–22.02) | 1.23 |
| 75–79 | 113 | 40 | 73 | 13.83 | (11.28–16.38) | 14.06 | (9.70–18.42) | 13.71 | (10.56–16.85) | 1.03 |
| 80–84 | 33 | 14 | 19 | 7.30 | (4.81–9.79) | 9.92 | (4.72–15.11) | 6.11 | (3.36–8.85) | 1.62 |
| 85–89 | 11 | 5 | 6 | 5.42 | (2.22–8.63) | 8.93 | (1.10–16.76) | 4.09 | (0.82–7.35) | 2.19 |
| 90–94 | NA | NA | NA | NA | NA | NA | NA | NA | NA |  |
| 95– | NA | NA | NA | NA | NA | NA | NA | NA | NA |  |
| Total | 4145 | 2256 | 1889 | 8.44 | (8.18–8.69) | 9.16 | (8.78–9.54) | 7.71 | (7.36–8.06) | 1.19 |
|  |  |  |  |  |  |  |  |  |  |  |
|  | **Standardized N of Cases** | | | **Standardized Incidence Rate (per 1,000,000 person-years)*** | | | | | |  |
|  | **Total** | **Male** | **Female** | **Total** | **95%CI** | **Male** | **95%CI** | **Female** | **95%CI** | **M:F Ratio** |
| Total | 4866.1 | 2645.5 | 2223.4 | 9.80 | (9.52–10.07) | 10.66 | (10.25–11.07) | 8.93 | (8.56–9.31) | 1.19 |

***In reference to the 2015 Census Data**

**NA; not available**

**Supplemental Table 18. Number of Cases of Rhegmatogenous Retinal Detachment (RRD) Requiring Surgery and Crude and Age- and Sex-Standardized Incidence Rate of RRD in 2008**

|  | **N of Cases** | | | **Crude Incidence Rate (per 1,000,000 person-years)** | | | | | |  |
| --- | --- | --- | --- | --- | --- | --- | --- | --- | --- | --- |
| **Age** | **Total** | **Male** | **Female** | **Total** | **95%CI** | **Male** | **95%CI** | **Female** | **95%CI** | **M:F Ratio** |
| 0–4 | NA | NA | NA | NA | NA | NA | NA | NA | NA | 0.00 |
| 5–9 | 10 | 7 | NA | 0.35 | (0.13–0.56) | 0.47 | (0.12–0.81) | NA | NA |  |
| 10–14 | 86 | 70 | 16 | 2.49 | (1.96–3.01) | 3.83 | (2.94–4.73) | 0.98 | (0.50–1.46) | 3.91 |
| 15–19 | 186 | 132 | 54 | 5.54 | (4.74–6.33) | 7.42 | (6.15–8.68) | 3.42 | (2.51–4.33) | 2.17 |
| 20–24 | 246 | 131 | 115 | 7.63 | (6.67–8.58) | 7.81 | (6.47–9.15) | 7.42 | (6.07–8.78) | 1.05 |
| 25–29 | 274 | 132 | 142 | 6.77 | (5.97–7.57) | 6.36 | (5.28–7.45) | 7.19 | (6.01–8.38) | 0.88 |
| 30–34 | 223 | 114 | 109 | 5.59 | (4.86–6.32) | 5.61 | (4.58–6.64) | 5.57 | (4.52–6.61) | 1.01 |
| 35–39 | 246 | 143 | 103 | 5.37 | (4.70–6.04) | 6.12 | (5.12–7.13) | 4.59 | (3.70–5.47) | 1.34 |
| 40–44 | 264 | 173 | 91 | 6.25 | (5.50–7.01) | 8.03 | (6.83–9.23) | 4.40 | (3.50–5.30) | 1.83 |
| 45–49 | 431 | 262 | 169 | 9.84 | (8.91–10.77) | 11.78 | (10.35–13.21) | 7.84 | (6.66–9.02) | 1.50 |
| 50–54 | 527 | 323 | 204 | 15.03 | (13.75–16.32) | 18.28 | (16.29–20.28) | 11.73 | (10.12–13.34) | 1.56 |
| 55–59 | 525 | 264 | 261 | 21.12 | (19.31–22.92) | 21.37 | (18.79–23.95) | 20.87 | (18.34–23.40) | 1.02 |
| 60–64 | 512 | 230 | 282 | 25.62 | (23.40–27.84) | 23.83 | (20.75–26.91) | 27.28 | (24.10–30.47) | 0.87 |
| 65–69 | 522 | 229 | 293 | 27.31 | (24.97–29.66) | 26.26 | (22.86–29.66) | 28.20 | (24.97–31.43) | 0.93 |
| 70–74 | 284 | 112 | 172 | 20.26 | (17.90–22.62) | 19.11 | (15.57–22.64) | 21.09 | (17.94–24.24) | 0.91 |
| 75–79 | 122 | 41 | 81 | 14.06 | (11.57–16.56) | 13.33 | (9.25–17.41) | 14.46 | (11.31–17.61) | 0.92 |
| 80–84 | 43 | 20 | 23 | 9.03 | (6.33–11.73) | 13.53 | (7.60–19.46) | 7.00 | (4.14–9.86) | 1.93 |
| 85–89 | 6 | NA | 5 | 2.67 | (0.53–4.80) | NA | NA | 3.07 | (0.38–5.76) |  |
| 90–94 | NA | NA | NA | NA | NA | NA | NA | NA | NA |  |
| 95– | NA | NA | NA | NA | NA | NA | NA | NA | NA |  |
| Total | 4508 | 2384 | 2124 | 9.12 | (8.86–9.39) | 9.63 | (9.24–10.02) | 8.62 | (8.25–8.98) | 1.12 |
|  |  |  |  |  |  |  |  |  |  |  |
|  | **Standardized N of Cases** | | | **Standardized Incidence Rate (per 1,000,000 person-years)*** | | | | | |  |
|  | **Total** | **Male** | **Female** | **Total** | **95%CI** | **Male** | **95%CI** | **Female** | **95%CI** | **M:F Ratio** |
| Total | 5150.9 | 2711.0 | 2436.1 | 10.36 | (10.08–10.64) | 10.92 | (10.51–11.33) | 9.79 | (9.40–10.18) | 1.12 |

***In reference to the 2015 Census Data**

**NA; not available**

**Supplemental Table 19. Number of Cases of Rhegmatogenous Retinal Detachment (RRD) Requiring Surgery and Crude and Age- and Sex-Standardized Incidence Rate of RRD in 2009**

|  | **N of Cases** | | | **Crude Incidence Rate (per 1,000,000 person-years)** | | | | | |  |
| --- | --- | --- | --- | --- | --- | --- | --- | --- | --- | --- |
| **Age** | **Total** | **Male** | **Female** | **Total** | **95%CI** | **Male** | **95%CI** | **Female** | **95%CI** | **M:F Ratio** |
| 0–4 | NA | NA | NA | NA | NA | NA | NA | NA | NA |  |
| 5–9 | 6 | 5 | NA | 0.22 | (0.04–0.40) | 0.35 | (0.04–0.66) | NA | NA |  |
| 10–14 | 89 | 67 | 22 | 2.64 | (2.09–3.19) | 3.78 | (2.87–4.68) | 1.37 | (0.80–1.95) | 2.75 |
| 15–19 | 202 | 147 | 55 | 5.86 | (5.06–6.67) | 8.03 | (6.74–9.33) | 3.41 | (2.51–4.31) | 2.36 |
| 20–24 | 221 | 127 | 94 | 7.02 | (6.09–7.94) | 7.73 | (6.38–9.07) | 6.24 | (4.98–7.50) | 1.24 |
| 25–29 | 267 | 128 | 139 | 6.71 | (5.91–7.52) | 6.28 | (5.19–7.36) | 7.17 | (5.98–8.36) | 0.88 |
| 30–34 | 228 | 113 | 115 | 5.86 | (5.10–6.62) | 5.70 | (4.65–6.75) | 6.04 | (4.93–7.14) | 0.94 |
| 35–39 | 240 | 141 | 99 | 5.30 | (4.63–5.97) | 6.10 | (5.09–7.11) | 4.46 | (3.58–5.34) | 1.37 |
| 40–44 | 309 | 200 | 109 | 7.13 | (6.34–7.93) | 9.08 | (7.82–10.34) | 5.12 | (4.16–6.08) | 1.77 |
| 45–49 | 403 | 263 | 140 | 9.22 | (8.32–10.12) | 11.83 | (10.40–13.27) | 6.52 | (5.44–7.60) | 1.81 |
| 50–54 | 546 | 313 | 233 | 14.60 | (13.38–15.83) | 16.61 | (14.77–18.45) | 12.57 | (10.95–14.18) | 1.32 |
| 55–59 | 490 | 258 | 232 | 18.95 | (17.27–20.63) | 20.07 | (17.62–22.52) | 17.85 | (15.55–20.14) | 1.12 |
| 60–64 | 537 | 275 | 262 | 25.73 | (23.55–27.90) | 27.08 | (23.88–30.28) | 24.45 | (21.49–27.41) | 1.11 |
| 65–69 | 435 | 197 | 238 | 22.67 | (20.54–24.80) | 22.41 | (19.28–25.54) | 22.89 | (19.98–25.80) | 0.98 |
| 70–74 | 293 | 133 | 160 | 19.80 | (17.54–22.07) | 21.24 | (17.63–24.84) | 18.75 | (15.85–21.66) | 1.13 |
| 75–79 | 135 | 50 | 85 | 14.58 | (12.12–17.04) | 14.93 | (10.79–19.07) | 14.39 | (11.33–17.44) | 1.04 |
| 80–84 | 49 | 19 | 30 | 9.57 | (6.89–12.26) | 12.02 | (6.61–17.42) | 8.48 | (5.45–11.52) | 1.42 |
| 85–89 | 9 | 6 | NA | 3.71 | (1.29–6.13) | 8.99 | (1.80–16.19) | NA | NA |  |
| 90–94 | NA | NA | NA | NA | NA | NA | NA | NA | NA |  |
| 95– | NA | NA | NA | NA | NA | NA | NA | NA | NA |  |
| Total | 4460 | 2442 | 2018 | 8.98 | (8.72–9.25) | 9.82 | (9.43–10.21) | 8.14 | (7.79–8.50) | 1.21 |
|  |  |  |  |  |  |  |  |  |  |  |
|  | **Standardized N of Cases** | | | **Standardized Incidence Rate (per 1,000,000 person-years)*** | | | | | |  |
|  | **Total** | **Male** | **Female** | **Total** | **95%CI** | **Male** | **95%CI** | **Female** | **95%CI** | **M:F Ratio** |
| Total | 4968.9 | 2714.4 | 2254.3 | 10.00 | (9.72–10.27) | 10.94 | (10.52–11.35) | 9.06 | (8.68–9.43) | 1.21 |

***In reference to the 2015 Census Data**

**NA; not available**

**Supplemental Table 20. Number of Cases of Rhegmatogenous Retinal Detachment (RRD) Requiring Surgery and Crude and Age- and Sex-Standardized Incidence Rate of RRD in 2010**

|  | **N of Cases** | | | **Crude Incidence Rate (per 1,000,000 person-years)** | | | | | |  |
| --- | --- | --- | --- | --- | --- | --- | --- | --- | --- | --- |
| **Age** | **Total** | **Male** | **Female** | **Total** | **95%CI** | **Male** | **95%CI** | **Female** | **95%CI** | **M:F Ratio** |
| 0–4 | NA | NA | NA | NA | NA | NA | NA | NA | NA |  |
| 5–9 | 12 | 10 | NA | 0.47 | (0.20–0.73) | 0.75 | (0.29–1.22) | NA | NA |  |
| 10–14 | 66 | 55 | 11 | 2.01 | (1.52–2.49) | 3.19 | (2.35–4.03) | 0.70 | (0.29–1.12) | 4.55 |
| 15–19 | 273 | 199 | 74 | 7.76 | (6.84–8.68) | 10.64 | (9.16–12.11) | 4.49 | (3.47–5.51) | 2.37 |
| 20–24 | 282 | 172 | 110 | 9.02 | (7.96–10.07) | 10.50 | (8.93–12.06) | 7.39 | (6.01–8.77) | 1.42 |
| 25–29 | 295 | 146 | 149 | 7.76 | (6.88–8.65) | 7.48 | (6.27–8.69) | 8.06 | (6.77–9.36) | 0.93 |
| 30–34 | 252 | 137 | 115 | 6.47 | (5.67–7.27) | 6.90 | (5.75–8.06) | 6.03 | (4.92–7.13) | 1.15 |
| 35–39 | 232 | 140 | 92 | 5.24 | (4.56–5.91) | 6.19 | (5.17–7.22) | 4.24 | (3.37–5.10) | 1.46 |
| 40–44 | 324 | 204 | 120 | 7.36 | (6.56–8.16) | 9.12 | (7.87–10.37) | 5.54 | (4.55–6.53) | 1.65 |
| 45–49 | 360 | 233 | 127 | 8.35 | (7.49–9.21) | 10.61 | (9.25–11.97) | 6.00 | (4.96–7.05) | 1.77 |
| 50–54 | 605 | 363 | 242 | 15.33 | (14.11–16.56) | 18.27 | (16.39–20.15) | 12.36 | (10.80–13.91) | 1.48 |
| 55–59 | 585 | 269 | 316 | 21.03 | (19.33–22.74) | 19.46 | (17.13–21.78) | 22.59 | (20.10–25.08) | 0.86 |
| 60–64 | 597 | 302 | 295 | 27.28 | (25.09–29.47) | 28.24 | (25.05–31.43) | 26.36 | (23.35–29.37) | 1.07 |
| 65–69 | 470 | 208 | 262 | 24.68 | (22.45–26.91) | 23.71 | (20.48–26.93) | 25.52 | (22.43–28.60) | 0.93 |
| 70–74 | 299 | 122 | 177 | 19.45 | (17.25–21.66) | 18.56 | (15.26–21.85) | 20.12 | (17.16–23.09) | 0.92 |
| 75–79 | 149 | 49 | 100 | 14.91 | (12.51–17.30) | 13.23 | (9.52–16.93) | 15.90 | (12.78–19.01) | 0.83 |
| 80–84 | 56 | 18 | 38 | 10.12 | (7.47–12.77) | 10.56 | (5.68–15.44) | 9.92 | (6.77–13.08) | 1.06 |
| 85–89 | 10 | NA | 6 | 3.90 | (1.48–6.31) | NA | NA | 3.21 | (0.64–5.79) |  |
| 90–94 | NA | NA | NA | NA | NA | NA | NA | NA | NA |  |
| 95– | NA | NA | NA | NA | NA | NA | NA | NA | NA |  |
| Total | 4870 | 2634 | 2236 | 9.76 | (9.49–10.04) | 10.55 | (10.14–10.95) | 8.98 | (8.61–9.35) | 1.17 |
|  |  |  |  |  |  |  |  |  |  |  |
|  | **Standardized N of Cases** | | | **Standardized Incidence Rate (per 1,000,000 person-years)*** | | | | | |  |
|  | **Total** | **Male** | **Female** | **Total** | **95%CI** | **Male** | **95%CI** | **Female** | **95%CI** | **M:F Ratio** |
| Total | 5313.2 | 2841.4 | 2467.7 | 10.68 | (10.39–10.97) | 11.45 | (11.03–11.87) | 9.92 | (9.52–10.31) | 1.15 |

***In reference to the 2015 Census Data**

**NA; not available**

**Supplemental Table 21. Number of Cases of Rhegmatogenous Retinal Detachment (RRD) Requiring Surgery and Crude and Age- and Sex-Standardized Incidence Rate of RRD in 2011**

|  | **N of Cases** | | | **Crude Incidence Rate (per 1,000,000 person-years)** | | | | | |  |
| --- | --- | --- | --- | --- | --- | --- | --- | --- | --- | --- |
| **Age** | **Total** | **Male** | **Female** | **Total** | **95%CI** | **Male** | **95%CI** | **Female** | **95%CI** | **M:F Ratio** |
| 0–4 | NA | NA | NA | NA | NA | NA | NA | NA | NA | 0.00 |
| 5–9 | 10 | 7 | NA | 0.42 | (0.16–0.67) | 0.56 | (0.15–0.98) | NA | NA |  |
| 10–14 | 53 | 41 | 12 | 1.66 | (1.21–2.11) | 2.46 | (1.71–3.21) | 0.79 | (0.34–1.23) | 3.12 |
| 15–19 | 226 | 165 | 61 | 6.38 | (5.54–7.21) | 8.77 | (7.43–10.10) | 3.67 | (2.75–4.59) | 2.39 |
| 20–24 | 253 | 137 | 116 | 8.01 | (7.02–8.99) | 8.25 | (6.87–9.63) | 7.74 | (6.33–9.15) | 1.07 |
| 25–29 | 303 | 153 | 150 | 8.42 | (7.47–9.37) | 8.26 | (6.95–9.57) | 8.59 | (7.21–9.96) | 0.96 |
| 30–34 | 263 | 139 | 124 | 6.65 | (5.85–7.46) | 6.89 | (5.75–8.04) | 6.40 | (5.28–7.53) | 1.08 |
| 35–39 | 259 | 144 | 115 | 6.06 | (5.32–6.80) | 6.61 | (5.53–7.70) | 5.48 | (4.48–6.49) | 1.21 |
| 40–44 | 284 | 156 | 128 | 6.32 | (5.59–7.06) | 6.85 | (5.77–7.92) | 5.78 | (4.78–6.78) | 1.18 |
| 45–49 | 376 | 235 | 141 | 8.92 | (8.02–9.82) | 10.90 | (9.51–12.29) | 6.84 | (5.71–7.97) | 1.59 |
| 50–54 | 562 | 353 | 209 | 13.57 | (12.45–14.70) | 16.93 | (15.16–18.70) | 10.17 | (8.79–11.55) | 1.67 |
| 55–59 | 548 | 289 | 259 | 18.06 | (16.54–19.57) | 19.18 | (16.96–21.39) | 16.95 | (14.89–19.02) | 1.13 |
| 60–64 | 556 | 289 | 267 | 24.55 | (22.51–26.59) | 26.09 | (23.08–29.09) | 23.07 | (20.31–25.84) | 1.13 |
| 65–69 | 397 | 186 | 211 | 21.06 | (18.98–23.13) | 21.23 | (18.18–24.29) | 20.90 | (18.08–23.72) | 1.02 |
| 70–74 | 264 | 119 | 145 | 16.59 | (14.59–18.59) | 17.33 | (14.21–20.44) | 16.04 | (13.43–18.65) | 1.08 |
| 75–79 | 125 | 46 | 79 | 11.57 | (9.54–13.60) | 11.23 | (7.98–14.47) | 11.78 | (9.18–14.37) | 0.95 |
| 80–84 | 55 | 20 | 35 | 9.29 | (6.83–11.74) | 10.94 | (6.14–15.73) | 8.55 | (5.72–11.39) | 1.28 |
| 85–89 | 17 | 7 | 10 | 6.28 | (3.30–9.27) | 9.60 | (2.49–16.71) | 5.06 | (1.92–8.20) | 1.90 |
| 90–94 | NA | NA | NA | NA | NA | NA | NA | NA | NA |  |
| 95– | NA | NA | NA | NA | NA | NA | NA | NA | NA |  |
| Total | 4555 | 2487 | 2068 | 9.09 | (8.83–9.35) | 9.92 | (9.53–10.31) | 8.26 | (7.91–8.62) | 1.20 |
|  |  |  |  |  |  |  |  |  |  |  |
|  | **Standardized N of Cases** | | | **Standardized Incidence Rate (per 1,000,000 person-years)*** | | | | | |  |
|  | **Total** | **Male** | **Female** | **Total** | **95%CI** | **Male** | **95%CI** | **Female** | **95%CI** | **M:F Ratio** |
| Total | 4830.6 | 2629.3 | 2200.0 | 9.72 | (9.44–9.99) | 10.59 | (10.19–11.00) | 8.84 | (8.47–9.21) | 1.20 |

***In reference to the 2015 Census Data**

**NA; not available**

**Supplemental Table 22. Number of Cases of Rhegmatogenous Retinal Detachment (RRD) Requiring Surgery and Crude and Age- and Sex-Standardized Incidence Rate of RRD in 2012**

|  | **N of Cases** | | | **Crude Incidence Rate (per 1,000,000 person-years)** | | | | | |  |
| --- | --- | --- | --- | --- | --- | --- | --- | --- | --- | --- |
| **Age** | **Total** | **Male** | **Female** | **Total** | **95%CI** | **Male** | **95%CI** | **Female** | **95%CI** | **M:F Ratio** |
| 0–4 | NA | NA | NA | NA | NA | NA | NA | NA | NA |  |
| 5–9 | 7 | 6 | NA | 0.30 | (0.08–0.52) | 0.49 | (0.10–0.89) | NA | NA |  |
| 10–14 | 71 | 53 | 18 | 2.34 | (1.79–2.88) | 3.34 | (2.44–4.24) | 1.24 | (0.67–1.81) | 2.70 |
| 15–19 | 217 | 151 | 66 | 6.18 | (5.36–7.01) | 8.12 | (6.83–9.42) | 4.00 | (3.04–4.97) | 2.03 |
| 20–24 | 330 | 186 | 144 | 10.15 | (9.05–11.24) | 10.85 | (9.29–12.41) | 9.36 | (7.83–10.89) | 1.16 |
| 25–29 | 263 | 124 | 139 | 7.76 | (6.82–8.69) | 7.09 | (5.84–8.34) | 8.46 | (7.06–9.87) | 0.84 |
| 30–34 | 242 | 130 | 112 | 6.03 | (5.27–6.80) | 6.35 | (5.26–7.44) | 5.71 | (4.65–6.76) | 1.11 |
| 35–39 | 248 | 145 | 103 | 6.02 | (5.27–6.76) | 6.92 | (5.79–8.05) | 5.08 | (4.10–6.06) | 1.36 |
| 40–44 | 297 | 180 | 117 | 6.50 | (5.77–7.24) | 7.77 | (6.63–8.90) | 5.20 | (4.26–6.15) | 1.49 |
| 45–49 | 365 | 235 | 130 | 8.80 | (7.90–9.70) | 11.08 | (9.66–12.50) | 6.41 | (5.31–7.52) | 1.73 |
| 50–54 | 577 | 328 | 249 | 13.45 | (12.35–14.55) | 15.18 | (13.54–16.82) | 11.70 | (10.24–13.15) | 1.30 |
| 55–59 | 607 | 330 | 277 | 18.69 | (17.20–20.18) | 20.42 | (18.22–22.62) | 16.97 | (14.98–18.97) | 1.20 |
| 60–64 | 542 | 277 | 265 | 23.16 | (21.21–25.11) | 24.17 | (21.33–27.02) | 22.18 | (19.51–24.85) | 1.09 |
| 65–69 | 378 | 183 | 195 | 20.14 | (18.11–22.17) | 20.80 | (17.79–23.82) | 19.55 | (16.81–22.29) | 1.06 |
| 70–74 | 294 | 137 | 157 | 17.37 | (15.39–19.36) | 18.56 | (15.46–21.67) | 16.45 | (13.88–19.03) | 1.13 |
| 75–79 | 133 | 59 | 74 | 11.53 | (9.57–13.49) | 13.25 | (9.87–16.63) | 10.45 | (8.07–12.83) | 1.27 |
| 80–84 | 41 | 13 | 28 | 6.47 | (4.49–8.45) | 6.55 | (2.99–10.11) | 6.43 | (4.05–8.82) | 1.02 |
| 85–89 | 10 | NA | 8 | 3.50 | (1.33–5.67) | NA | NA | 3.82 | (1.17–6.47) |  |
| 90–94 | NA | NA | NA | NA | NA | NA | NA | NA | NA |  |
| 95– | NA | NA | NA | NA | NA | NA | NA | NA | NA |  |
| Total | 4625 | 2541 | 2084 | 9.19 | (8.92–9.45) | 10.09 | (9.70–10.48) | 8.28 | (7.93–8.64) | 1.22 |
|  |  |  |  |  |  |  |  |  |  |  |
|  | **Standardized N of Cases** | | | **Standardized Incidence Rate (per 1,000,000 person-years)*** | | | | | |  |
|  | **Total** | **Male** | **Female** | **Total** | **95%CI** | **Male** | **95%CI** | **Female** | **95%CI** | **M:F Ratio** |
| Total | 4809.0 | 2636.6 | 2171.9 | 9.67 | (9.40–9.95) | 10.62 | (10.22–11.03) | 8.73 | (8.36–9.09) | 1.22 |

***In reference to the 2015 Census Data**

**NA; not available Supplemental Table 23. Number of Cases of Rhegmatogenous Retinal Detachment (RRD) Requiring Surgery and Crude and Age- and Sex-Standardized Incidence Rate of RRD in 2013**

|  | **N of Cases** | | | **Crude Incidence Rate (per 1,000,000 person-years)** | | | | | |  |
| --- | --- | --- | --- | --- | --- | --- | --- | --- | --- | --- |
| **Age** | **Total** | **Male** | **Female** | **Total** | **95%CI** | **Male** | **95%CI** | **Female** | **95%CI** | **M:F Ratio** |
| 0–4 | NA | NA | NA | NA | NA | NA | NA | NA | NA |  |
| 5–9 | NA | NA | NA | NA | NA | NA | NA | NA | NA | 0.93 |
| 10–14 | 40 | 30 | 10 | 1.39 | (0.96–1.82) | 2.00 | (1.28–2.71) | 0.73 | (0.28–1.18) | 2.75 |
| 15–19 | 223 | 157 | 66 | 6.47 | (5.62–7.32) | 8.63 | (7.28–9.98) | 4.06 | (3.08–5.03) | 2.13 |
| 20–24 | 296 | 155 | 141 | 8.84 | (7.83–9.85) | 8.75 | (7.38–10.13) | 8.93 | (7.46–10.41) | 0.98 |
| 25–29 | 227 | 106 | 121 | 7.03 | (6.12–7.95) | 6.36 | (5.15–7.57) | 7.76 | (6.38–9.15) | 0.82 |
| 30–34 | 260 | 149 | 111 | 6.43 | (5.65–7.21) | 7.22 | (6.06–8.38) | 5.61 | (4.57–6.66) | 1.29 |
| 35–39 | 249 | 145 | 104 | 6.26 | (5.48–7.04) | 7.17 | (6.01–8.34) | 5.32 | (4.30–6.34) | 1.35 |
| 40–44 | 353 | 233 | 120 | 7.74 | (6.93–8.55) | 10.04 | (8.75–11.33) | 5.36 | (4.40–6.31) | 1.88 |
| 45–49 | 437 | 286 | 151 | 10.41 | (9.44–11.39) | 13.41 | (11.86–14.97) | 7.32 | (6.15–8.49) | 1.83 |
| 50–54 | 687 | 421 | 266 | 15.84 | (14.66–17.03) | 19.24 | (17.41–21.08) | 12.38 | (10.89–13.87) | 1.55 |
| 55–59 | 695 | 389 | 306 | 20.15 | (18.65–21.64) | 22.60 | (20.35–24.84) | 17.70 | (15.72–19.69) | 1.28 |
| 60–64 | 553 | 307 | 246 | 22.82 | (20.92–24.72) | 25.87 | (22.97–28.76) | 19.90 | (17.41–22.38) | 1.30 |
| 65–69 | 445 | 211 | 234 | 23.17 | (21.02–25.33) | 23.24 | (20.10–26.37) | 23.12 | (20.16–26.08) | 1.01 |
| 70–74 | 334 | 156 | 178 | 18.85 | (16.82–20.87) | 20.02 | (16.88–23.17) | 17.92 | (15.29–20.55) | 1.12 |
| 75–79 | 154 | 52 | 102 | 12.60 | (10.61–14.59) | 10.84 | (7.90–13.79) | 13.73 | (11.07–16.40) | 0.79 |
| 80–84 | 68 | 23 | 45 | 10.02 | (7.64–12.41) | 10.58 | (6.26–14.91) | 9.76 | (6.91–12.61) | 1.08 |
| 85–89 | 12 | NA | 8 | 3.92 | (1.70–6.14) | NA | NA | 3.56 | (1.09–6.04) |  |
| 90–94 | NA | NA | NA | NA | NA | NA | NA | NA | NA |  |
| 95– | NA | NA | NA | NA | NA | NA | NA | NA | NA |  |
| Total | 5037 | 2826 | 2211 | 9.96 | (9.69–10.24) | 11.18 | (10.77–11.59) | 8.75 | (8.38–9.11) | 1.28 |
|  |  |  |  |  |  |  |  |  |  |  |
|  | **Standardized N of Cases** | | | **Standardized Incidence Rate (per 1,000,000 person-years)*** | | | | | |  |
|  | **Total** | **Male** | **Female** | **Total** | **95%CI** | **Male** | **95%CI** | **Female** | **95%CI** | **M:F Ratio** |
| Total | 5148.9 | 2878.8 | 2267.8 | 10.35 | (10.07–10.64) | 11.60 | (11.18–12.02) | 9.11 | (8.74–9.49) | 1.27 |

***In reference to the 2015 Census Data**

**NA; not available**

**Supplemental Table 24. Number of Cases of Rhegmatogenous Retinal Detachment (RRD) Requiring Surgery and Crude and Age- and Sex-Standardized Incidence Rate of RRD in 2014**

|  | **N of Cases** | | | **Crude Incidence Rate (per 1,000,000 person-years)** | | | | | |  |
| --- | --- | --- | --- | --- | --- | --- | --- | --- | --- | --- |
| **Age** | **Total** | **Male** | **Female** | **Total** | **95%CI** | **Male** | **95%CI** | **Female** | **95%CI** | **M:F Ratio** |
| 0–4 | NA | NA | NA | NA | NA | NA | NA | NA | NA |  |
| 5–9 | 8 | 7 | NA | 0.35 | (0.11–0.59) | 0.59 | (0.15–1.02) | NA | NA |  |
| 10–14 | 50 | 35 | 15 | 1.83 | (1.32–2.34) | 2.46 | (1.64–3.27) | 1.15 | (0.57–1.73) | 2.14 |
| 15–19 | 234 | 161 | 73 | 6.96 | (6.06–7.85) | 9.11 | (7.70–10.51) | 4.57 | (3.52–5.62) | 1.99 |
| 20–24 | 289 | 156 | 133 | 8.42 | (7.45–9.39) | 8.57 | (7.22–9.91) | 8.25 | (6.84–9.65) | 1.04 |
| 25–29 | 215 | 130 | 85 | 6.82 | (5.91–7.73) | 7.95 | (6.59–9.32) | 5.60 | (4.41–6.79) | 1.42 |
| 30–34 | 278 | 162 | 116 | 7.00 | (6.18–7.82) | 7.99 | (6.76–9.22) | 5.97 | (4.88–7.06) | 1.34 |
| 35–39 | 257 | 143 | 114 | 6.63 | (5.82–7.44) | 7.25 | (6.06–8.44) | 5.99 | (4.89–7.09) | 1.21 |
| 40–44 | 376 | 229 | 147 | 8.34 | (7.50–9.18) | 9.98 | (8.68–11.27) | 6.64 | (5.57–7.71) | 1.50 |
| 45–49 | 501 | 326 | 175 | 11.65 | (10.63–12.67) | 14.96 | (13.34–16.59) | 8.25 | (7.02–9.47) | 1.81 |
| 50–54 | 646 | 350 | 296 | 14.95 | (13.79–16.10) | 16.02 | (14.34–17.70) | 13.85 | (12.27–15.43) | 1.16 |
| 55–59 | 783 | 422 | 361 | 21.29 | (19.80–22.78) | 22.98 | (20.79–25.17) | 19.60 | (17.58–21.62) | 1.17 |
| 60–64 | 606 | 307 | 299 | 24.04 | (22.12–25.95) | 24.83 | (22.06–27.61) | 23.27 | (20.63–25.91) | 1.07 |
| 65–69 | 457 | 220 | 237 | 22.75 | (20.66–24.84) | 22.96 | (19.93–26.00) | 22.56 | (19.68–25.43) | 1.02 |
| 70–74 | 306 | 137 | 169 | 17.14 | (15.22–19.06) | 17.36 | (14.45–20.26) | 16.97 | (14.41–19.53) | 1.02 |
| 75–79 | 177 | 77 | 100 | 13.65 | (11.64–15.67) | 14.92 | (11.59–18.25) | 12.82 | (10.30–15.33) | 1.16 |
| 80–84 | 60 | 24 | 36 | 8.22 | (6.14–10.30) | 10.02 | (6.01–14.03) | 7.34 | (4.94–9.74) | 1.36 |
| 85–89 | 18 | 7 | 11 | 5.39 | (2.90–7.89) | 7.89 | (2.04–13.73) | 4.49 | (1.84–7.14) | 1.76 |
| 90–94 | NA | NA | NA | NA | NA | NA | NA | NA | NA |  |
| 95– | NA | NA | NA | NA | NA | NA | NA | NA | NA |  |
| Total | 5262 | 2893 | 2369 | 10.37 | (10.09–10.65) | 11.40 | (10.99–11.82) | 9.33 | (8.96–9.71) | 1.22 |
|  |  |  |  |  |  |  |  |  |  |  |
|  | **Standardized N of Cases** | | | **Standardized Incidence Rate (per 1,000,000 person-years)*** | | | | | |  |
|  | **Total** | **Male** | **Female** | **Total** | **95%CI** | **Male** | **95%CI** | **Female** | **95%CI** | **M:F Ratio** |
| Total | 5278.1 | 2892.9 | 2384.7 | 10.62 | (10.33–10.90) | 11.66 | (11.23–12.08) | 9.58 | (9.20–9.97) | 1.22 |

***In reference to the 2015 Census Data**

**NA; not available**

**Supplemental Table 25. Number of Cases of Rhegmatogenous Retinal Detachment (RRD) Requiring Surgery and Crude and Age- and Sex-Standardized Incidence Rate of RRD in 2015**

|  | **N of Cases** | | | **Crude Incidence Rate (per 1,000,000 person-years)** | | | | | |  |
| --- | --- | --- | --- | --- | --- | --- | --- | --- | --- | --- |
| **Age** | **Total** | **Male** | **Female** | **Total** | **95%CI** | **Male** | **95%CI** | **Female** | **95%CI** | **M:F Ratio** |
| 0–4 | NA | NA | NA | NA | NA | NA | NA | NA | NA |  |
| 5–9 | 7 | NA | NA | 0.30 | (0.08–0.53) | NA | NA | NA | NA |  |
| 10–14 | 47 | 35 | 12 | 1.84 | (1.31–2.36) | 2.63 | (1.76–3.50) | 0.98 | (0.42–1.53) | 2.69 |
| 15–19 | 212 | 151 | 61 | 6.46 | (5.59–7.33) | 8.79 | (7.39–10.19) | 3.90 | (2.92–4.88) | 2.25 |
| 20–24 | 270 | 165 | 105 | 7.69 | (6.78–8.61) | 8.86 | (7.51–10.21) | 6.37 | (5.16–7.59) | 1.39 |
| 25–29 | 225 | 111 | 114 | 7.19 | (6.25–8.13) | 6.81 | (5.54–8.07) | 7.60 | (6.21–9.00) | 0.90 |
| 30–34 | 228 | 125 | 103 | 6.01 | (5.23–6.79) | 6.43 | (5.31–7.56) | 5.56 | (4.48–6.63) | 1.16 |
| 35–39 | 252 | 151 | 101 | 6.49 | (5.69–7.29) | 7.65 | (6.43–8.87) | 5.30 | (4.26–6.33) | 1.44 |
| 40–44 | 335 | 214 | 121 | 7.60 | (6.78–8.41) | 9.53 | (8.26–10.81) | 5.59 | (4.59–6.58) | 1.71 |
| 45–49 | 490 | 296 | 194 | 11.21 | (10.21–12.20) | 13.37 | (11.84–14.89) | 8.99 | (7.72–10.25) | 1.49 |
| 50–54 | 717 | 459 | 258 | 16.81 | (15.58–18.04) | 21.25 | (19.31–23.20) | 12.25 | (10.75–13.74) | 1.73 |
| 55–59 | 815 | 450 | 365 | 20.99 | (19.55–22.43) | 23.23 | (21.08–25.37) | 18.76 | (16.84–20.69) | 1.24 |
| 60–64 | 689 | 364 | 325 | 25.38 | (23.48–27.27) | 27.33 | (24.53–30.14) | 23.49 | (20.94–26.05) | 1.16 |
| 65–69 | 505 | 255 | 250 | 23.94 | (21.85–26.03) | 25.23 | (22.13–28.32) | 22.76 | (19.94–25.58) | 1.11 |
| 70–74 | 325 | 151 | 174 | 18.29 | (16.30–20.27) | 19.07 | (16.02–22.11) | 17.66 | (15.03–20.28) | 1.08 |
| 75–79 | 195 | 88 | 107 | 14.41 | (12.38–16.43) | 16.12 | (12.75–19.49) | 13.25 | (10.74–15.76) | 1.22 |
| 80–84 | 64 | 26 | 38 | 8.06 | (6.08–10.03) | 9.71 | (5.98–13.44) | 7.22 | (4.92–9.51) | 1.34 |
| 85–89 | 29 | 10 | 19 | 7.94 | (5.05–10.83) | 10.31 | (3.92–16.70) | 7.09 | (3.90–10.28) | 1.45 |
| 90–94 | NA | NA | NA | NA | NA | NA | NA | NA | NA |  |
| 95– | NA | NA | NA | NA | NA | NA | NA | NA | NA |  |
| Total | 5408 | 3056 | 2352 | 10.61 | (10.33–10.90) | 12.00 | (11.58–12.43) | 9.23 | (8.85–9.60) | 1.30 |
|  |  |  |  |  |  |  |  |  |  |  |
|  | **Standardized N of Cases** | | | **Standardized Incidence Rate (per 1,000,000 person-years)*** | | | | | |  |
|  | **Total** | **Male** | **Female** | **Total** | **95%CI** | **Male** | **95%CI** | **Female** | **95%CI** | **M:F Ratio** |
| Total | 5325.0 | 3002.6 | 2321.5 | 10.71 | (10.42–11.00) | 12.10 | (11.67–12.53) | 9.33 | (8.95–9.71) | 1.30 |

***In reference to the 2015 Census Data**

**NA; not available**
